# Monocyte NLRP3‐IL‐1*β* Hyperactivation Mediates Neuronal and Synaptic Dysfunction in Perioperative Neurocognitive Disorder

**DOI:** 10.1002/advs.202104106

**Published:** 2022-03-28

**Authors:** Kai Chen, Qiuping Hu, Zhongcong Xie, Guang Yang

**Affiliations:** ^1^ Department of Anesthesiology Columbia University Irving Medical Center New York NY 10032 USA; ^2^ Geriatric Anesthesia Research Unit, Department of Anesthesia, Critical Care and Pain Medicine Massachusetts General Hospital and Harvard Medical School Charlestown MA 02129 USA

**Keywords:** IL‐1*β*, inflammasome, learning, monocytes, synaptic plasticity

## Abstract

Perioperative neurocognitive disorder may develop in vulnerable patients following major operation. While neuroinflammation is linked to the cognitive effects of surgery, how surgery and immune signaling modulate neuronal circuits, leading to learning and memory impairment remains unknown. Using in vivo two‐photon microscopy, Ca^2+^ activity and postsynaptic dendritic spines of layer 5 pyramidal neurons in the primary motor cortex of a mouse model of thoracic surgery are imaged. It is found that surgery causes neuronal hypoactivity, impairments in learning‐dependent dendritic spine formation, and deficits in multiple learning tasks. These neuronal and synaptic alterations in the cortex are mediated by peripheral monocytes through the NLRP3 inflammasome‐dependent IL‐1*β* production. Depleting peripheral monocytes or inactivating NLRP3 inflammasomes before surgery reduces levels of IL‐1*β* and ameliorates neuronal and behavioral deficits in mice. Furthermore, adoptive transfer of IL‐1*β*‐producing myeloid cells from mice undertaking thoracic surgery is sufficient to induce neuronal and behavioral deficits in naïve mice. Together, these findings suggest that surgery leads to excessive NLRP3 activation in monocytes and elevated IL‐1*β* signaling, which in turn causes neuronal hypoactivity and perioperative neurocognitive disorder.

## Graphical abstract

Perioperative neurocognitive disorder may develop in vulnerable patients following major surgery. How surgery‐induced inflammatory responses modulate neuronal circuits and thereby affect cognitive functioning remains unclear. Using in vivo imaging and cell type‐specific manipulation in mice, this study reveals that peripheral monocytes mediate neuronal and synaptic deficits after surgery through excessive NLRP3 activation and elevated IL‐1*β* signaling.

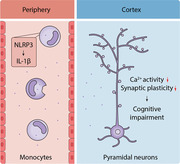

## 1 Introduction

In the US, an estimated 60 000 patients per day receive surgery under general anesthesia.^[^ ^1^ ^]^ Although most patients quickly regain their cognitive processing, a significant portion of previously cognitively well patients develops perioperative neurocognitive disorders (PNDs), which impair postoperative recovery, increase hospital stay and mortality.^[^ ^2^, ^3^, ^4^, ^5^, ^6^ ^]^ The symptoms of PND include impairments in learning, memory, and psychomotor function, and its duration may vary from days to years. Although more common and severe in elderly patients (>60 years), PND can occur in patients at any ages.^[^ ^7^, ^8^, ^9^ ^]^ Currently, there are no effective strategies to prevent or treat this surgical complication due to limited understanding of its pathophysiology.

Surgical injury is known to initiate a complex immune response.^[^ ^10^ ^]^ In patients, major surgery has been shown to cause a redistribution of immune cell types in peripheral blood, including a rapid expansion of neutrophils and CD14^+^ monocytes, and a contraction of CD4^+^ and CD8^+^ T cells at 24 and 72 h.^[^ ^11^ ^]^ Analysis of global gene expression in human circulating leukocytes 24 h after surgery revealed an upregulation of genes related to innate immune signaling and a downregulation of adaptive immune responses.^[^ ^12^ ^]^ Upon activation, innate immune cells initiate inflammatory pathways, including the nuclear factor‐*κ*B signaling, that increase the synthesis and release of cytokines such as tumor necrosis factor (TNF)‐*α*, interleukin (IL)‐1*β* and IL‐6. The resultant systemic and neuroinflammation has been associated with cognitive changes after surgery.^[^ ^13^, ^14^, ^15^, ^16^ ^]^ Previous studies in rodents showed that surgery‐induced memory impairment could be mitigated either by depleting bone marrow‐derived macrophages,^[^ ^17^, ^18^ ^]^ or by targeting microglia, the major innate immune cells in the brain.^[^ ^19^, ^20^, ^21^, ^22^ ^]^ However, how innate immune signaling modulates neuronal circuits, leading to impaired cognitive ability after surgery remains unknown.

Inflammasomes are signaling complexes that regulate the initiation and maintenance of innate immunity.^[^ ^23^ ^]^ The most extensively studied one in sterile inflammation is the NLRP3 inflammasome, which consists of NLRP3 (NOD‐, LRR‐, and pyrin domain‐containing protein 3), an adaptor protein ASC, and caspase‐1. As a cytosolic sensor, NLRP3 detects a broad array of pathogen patterns as well as endogenous danger signals released upon cellular stress or damage.^[^ ^24^ ^]^ The assembly of the NLRP3 inflammasome leads to the formation of active caspase‐1, which processes pro‐forms of proinflammatory cytokines IL‐1*β* and IL‐18 into mature forms. Dysregulation of the NLRP3 signaling has been implicated in a variety of inflammatory, neurodegenerative, and metabolic diseases such as atherosclerosis, Alzheimer's disease, Parkinson's disease, and diabetes.^[^ ^25^, ^26^ ^]^ However, whether activation of the NLRP3 inflammasome is involved in surgery‐induced cerebral dysfunction is not known.

Thoracic surgery is a common surgical procedure^[^ ^27^ ^]^ and has been reported to cause detectable changes in attention and psychomotor function in human patients.^[^ ^28^, ^29^ ^]^ In this study, we examined the alteration of cortical neurons and the role of innate immune signaling in a mouse model of thoracic surgery. Using in vivo two‐photon imaging, we showed that surgery causes a decrease of cortical neuronal activity during motor learning, paralleled by the reduction of learning‐dependent synapse formation and impaired performance in learning and memory tasks. We found that these surgery‐induced synaptic, neuronal, and behavioral changes are mediated by peripheral monocytes through the NLRP3 inflammasome and IL‐1*β*‐dependent mechanisms. Depleting monocytes or inhibiting NLRP3 inflammasomes prevents PND in mice.

## 2 Results

### 2.1 Surgery Causes Learning and Memory Impairment in Mice

Given that PND can affect patients of any age,^[^ ^9^ ^]^ we evaluated the effect of surgery on learning and memory in young (postnatal day 28, P28) and aged (>18 months) mice. Young mice were subjected to thoracic surgery under isoflurane anesthesia (hereafter referred to as surgery) on P28 and were tested on P29–30 and P34–35, respectively, for the short‐ and long‐term effects on behavior. Age‐matched control mice received no surgery and anesthesia. A separate group of mice received isoflurane anesthesia only.

**Figure 1:**
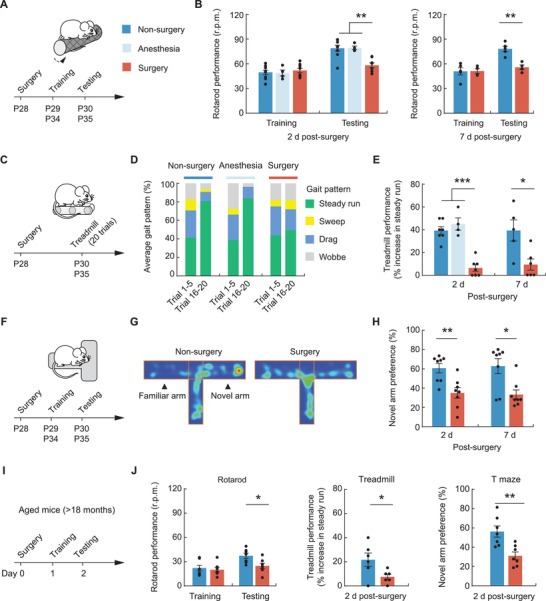
Surgery causes learning and memory impairment in both young and aged mice. A) Experimental timeline. Following thoracic surgery on P28, mice were tested in the rotarod task during P29–P30 or during P34–P35. B) Measures of rotarod performance expressed as the average speed reached during the training/testing session (*n* = 4–9 mice per group). C) Timeline for surgery and treadmill test. D) Analysis of the animals’ gait patterns when running on a treadmill 2 days after surgery. E) Measures of treadmill performance which is expressed as percent difference in steady run between the first and the last five running trials. F) Timeline for surgery and T maze test. G) Representative heatmap showing the accumulative time spent in each area of T maze. H) Percentage of time spent in the novel arm during a T maze test (*n* = 8 mice per group). I) Timeline for surgery and behavioral testing in aged mice (>18 months old). J) Performance of aged mice in rotarod, treadmill, and T maze tests (*n* = 6–7 mice per group). Throughout, each dots represent data from a single mouse. Summary data are presented as mean ± SEM. *p < 0.05, **p < 0.01, ***p < 0.001; by one‐way ANOVA followed by Bonferroni's post hoc test (B, E), Mann–Whitney test (H, J). See also Table S1 in the Supporting Information.

We first assessed the animals’ grooming and locomotor activity in an open field test (Figure S1, Supporting Information). 1 day after surgery (P29), mice in surgery group showed no signs of untreated pain or anxiety‐like behavior, as indicated by the measures of self‐grooming episodes and time spent in the center of open field arena. There was also no difference in the average speed and total distance traveled among three groups, indicating that surgery and anesthesia has no apparent impact on the animals’ locomotor function. Consistently, all groups of mice exhibited comparable performance in the rotarod task when they were first trained on P29 (**Figure** **1**A,B). However, when these mice were tested on the rotarod next day (P30) to examine their motor learning ability, we found that surgery group showed significant worse performance as compared to the other two groups. One week later (P34–35), mice in surgery group continued to show impaired motor learning as compared to nonsurgery controls (Figure 1A,B). In addition to the rotarod, we tested mice in another motor learning paradigm, treadmill running, where mice learned to adjust their gait feature (Figure 1C).^[^ ^30^ ^]^ In this treadmill task, mice displayed large proportions of drag, wobble, and sweep when they first ran on the treadmill, whereas the fraction of steady run increased over time with training (Figure 1D). Similar to the rotarod test, 2 and 7 days after surgery, we observed significant less increase in steady run in surgery mice versus nonsurgery mice, indicating impaired motor learning ability (Figure 1E).

To further investigate the effect of surgery on learning and memory, we examined mice in a T maze test. 1 or 6 days after surgery, mice were trained in the T maze and tested 1 day later to examine the animals’ spatial memory (Figure 1F). Compared to nonsurgery mice, we found mice with surgery spent less time exploring the novel arm in the testing session (Figure 1G,H and Figure S2, Supporting Information), indicating impaired spatial memory that persists for at least 1 week.

Like young mice, aged mice with surgery exhibited decreasing levels of performance in the rotarod, treadmill, and T maze tests as compared to age‐matched controls (Figure 1I,J and Figure S3, Supporting Information). Taken together, these results show that thoracic surgery causes learning and memory impairment in both young and aged mice.

### 2.2 Surgery Impairs Cortical Neuronal Activity Associated with Learning

**Figure 2:**
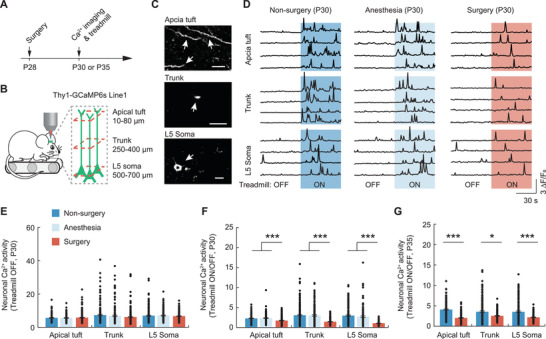
Learning‐dependent neuronal activity is decreased after surgery. A) Experimental timeline. Following surgery on P28, mice were imaged on either P30 or P35. B) Schematic illustrating in vivo Ca^2+^ imaging of L5 pyramidal neurons in the primary motor cortex of awake mice running on a treadmill. C) Examples of Ca^2+^ images acquired from the tuft, trunk, and soma of L5 neurons (indicated by arrows) in a P30 mouse. Scale bar, 30 µm. D) Ca^2+^ fluorescence traces of apical tuft dendrites, apical trunk dendrites, and L5 somas. Examples of 2 min traces are shown. E) Average Ca^2+^ activity in tuft/trunk dendrites and somas of P30 mice during resting. F) Fold change in Ca^2+^ activity in tuft/trunk dendrites and somas of P30 mice during running compared to rest (4–6 mice per group). G) Fold change in Ca^2+^ activity in tuft/trunk dendrites and somas of P35 mice during running compared to rest. Throughout, each dots represent data from a single dendrite or soma. Summary data are presented as mean ± SEM. *p < 0.05, ***p < 0.001; by one‐way ANOVA followed by Bonferroni's post hoc test (E, F), Mann–Whitney test (G). See also Table S1 in the Supporting Information.

Previous studies have suggested that activation of layer 5 (L5) pyramidal neurons during treadmill running is learning‐dependent.^[^ ^31^, ^32^ ^]^ To identify brain changes associated with motor learning impairment caused by surgery, we examined neuronal activity in the primary motor cortex of awake, head‐restrained mice using in vivo two‐photon Ca^2+^ imaging. Transgenic mice expressing genetically encoded calcium indicator GCaMP6 slow (GCaMP6s) in L5 pyramidal neurons were subjected to surgery on P28. 2 or 7 days after surgery, mice were head‐fixed and trained to run forward on a custom‐built treadmill under a two‐photon microscope (**Figure** **2**A). We recorded Ca^2+^ signals from different compartments of L5 neurons, including apical tuft dendrites (10–80 µm below the pial), apical trunk dendrites (250–400 µm below the pial), and somas (500–700 µm below the pial), during the quiet resting (i.e., 60 s treadmill OFF) and forward running (i.e., 60 s treadmill ON) (Figure 2B–D). At P30, when treadmill was off, the spontaneous Ca^2+^ activity in the apical tuft/trunk dendrites and somas of L5 neurons was similar across groups of mice with or without anesthesia or surgery (Figure 2E). When treadmill was on, there was a significant elevation of dendritic and somatic Ca^2+^ activity in all groups of mice, though to different extents (Figure 2D). Compared to the nonsurgery control group, we found that the level of running‐evoked Ca^2+^ activity was comparable in mice that received anesthesia only, but significantly lower in mice that received surgery (Apical tuft: nonsurgery, 2.30 ± 0.07, anesthesia, 2.36 ± 0.10, surgery, 1.74 ± 0.06; Trunk: nonsurgery, 3.07 ± 0.15, anesthesia, 3.01 ± 0.17, surgery, 1.46 ± 0.04; Soma: nonsurgery, 2.99 ± 0.15, anesthesia, 2.72 ± 0.19, surgery, 1.08 ± 0.04; one‐way analysis of variance (ANOVA) followed by Bonferroni's post hoc test, *p* < 0.0001) (Figure 2F and Figure S4, Supporting Information). 1 week after surgery (P35), mice continued to display a lower level of neuronal Ca^2+^ activity in the treadmill task as compared to the other groups (Figure 2G). Together, these results indicate that surgery in young mice causes a persistent reduction of cortical neuronal activity associated with motor learning.

### 2.3 Monocytes Are Required for Surgery‐Induced Neuronal and Learning Deficits

**Figure 3:**
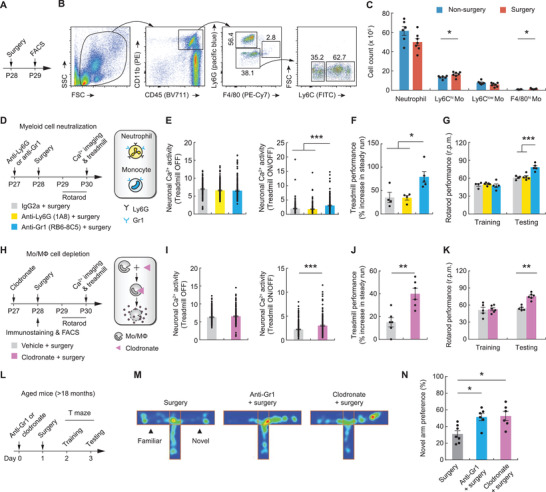
Depletion of monocytes prevents surgery‐induced neuronal and learning deficits. A) Experimental timeline for surgery and fluorescence‐activated cell sorting (FACS) analysis. B) Representative FACS analysis of blood cells. SSC, side scatter; FSC, forward scatter. C) Quantification of neutrophils, Ly6C^hi^ and Ly6C^low^ monocytes, and F4/80^hi^ monocytes in the blood (*n* = 6–7 mice per group). D) Experimental timeline and schematic of the strategy to deplete neutrophils and monocytes with anti‐Gr1 antibody, RB6‐8C5, or to selectively deplete neutrophils with anti‐Ly6G antibody, 1A8. E) Left, neuronal Ca^2+^ activity in the motor cortex when mice were resting. Right, fold change in neuronal Ca^2+^ activity during treadmill running versus rest (5 mice per group). F) Treadmill performance. G) Rotarod performance. H) Timeline and strategy to deplete monocytes/macrophages with clodronate. I–K) Similar to (E)–(G), but for mice with and without monocyte/macrophage depletion. L) Timeline for monocyte/macrophage depletion and behavioral testing in aged mice. M) Representative heatmap illustrating duration of time spent at each location in T maze. N) Percentage of time spent in the novel arm of T maze (*n* = 6–7 mice per group). Summary data are presented as mean ± SEM. E,I) Each dot represents data from a single neuron. C,F,G,J,K,N) Each dot represents data from a single mouse. *p < 0.05, **p < 0.01, ***p < 0.001; by one‐way ANOVA followed by Bonferroni's post hoc test (E–G, N), Mann–Whitney test (C, I–K). See also Table S1 in the Supporting Information.

We next investigated whether the observed effects of surgery on learning and learning‐evoked neuronal activity require the involvement of immune cells. Using immunostaining, we first examined the resident and infiltrating macrophages in the brain (Figure S5A, Supporting Information). 1 day after surgery, we observed no difference in the density and morphology of microglia in the motor cortex, as evidenced by the measures of Iba1^+^ cell density, soma size, mean fluorescence intensity, and major branch number (Figure S5B,C, Supporting Information). Immunostaining of CD206, a marker of macrophages,^[^ ^33^ ^]^ revealed no difference in the number of meningeal and parenchyma macrophages between surgery and nonsurgery groups (Figure S5D,E, Supporting Information), suggesting no infiltrating macrophages in the mouse cortex 24 h after surgery. Using flow cytometry, we characterized the frequency of circulating leukocytes in blood at 24 h. There was no difference in the number of neutrophils, T and B lymphocytes between surgery and nonsurgery groups (Figure S5F,G, Supporting Information), while Ly6C^hi^ and F4/80^hi^ monocytes were detected at a higher frequency in surgery group (**Figure** **3**A–C).

To determine whether peripheral monocytes contribute to neuronal and behavioral deficits after surgery, we performed in vivo cell depletion using antibody‐based strategies.^[^ ^34^, ^35^ ^]^ Specifically, mice were intravenously administered with anti‐granulocyte receptor‐1 (Gr‐1) monoclonal antibody (mAb), RB6‐8C5, which eliminates both Ly6G^+^ cells (e.g., neutrophils) and Ly6C^+^ cells (e.g., monocytes), or Ly6G‐specific mAb, 1A8, which selectively eliminates neutrophils (Figure 3D). Nondepleted control group received IgG2a injection. Following antibody treatment on P27, mice were subjected to surgery on P28. 2 days after surgery, we found that mice administered RB6‐8C5 showed a higher level of neuronal activity during treadmill running as compared to IgG2a‐treated controls. In contrast, administration of 1A8 had no significant effects on running‐evoked neuronal activity in surgery mice (Figure 3E). Consistent with the changes of neuronal activity, mice treated with RB6‐8C5 showed better performance in the treadmill and rotarod tasks than control mice, while 1A8 treatment had no significant effects on the animals’ motor learning performance (Figure 3F,G). These data suggest that Ly6C^+^ cells, but not neutrophils, contribute to the neuronal and learning deficits after surgery.

To validate the role of peripheral monocytes in surgery‐induced neuronal and behavioral deficits, we selectively depleted monocytes in vivo via intravenous administration of clodronate liposomes, which have been shown to cause apoptosis in monocytes and macrophages (Figure 3H).^[^ ^36^ ^]^ Using flow cytometry, we confirmed that the number of circulating monocytes was substantially decreased 1 day after the clodronate liposome administration (Figure S6A,B, Supporting Information). Consistent with previous reports that clodronate liposomes do not cross the blood–brain barrier,^[^ ^37^ ^]^ microglia in the cortex were largely intact after clodronate treatment (Figure S6C,D, Supporting Information), supporting the notion that clodronate selectively depletes peripheral monocytes.^[^ ^38^ ^]^ When mice depleted of monocytes were subjected to surgery, we found a significant increase in cortical neuronal activity during treadmill learning as compared to nondepleted mice treated with the vehicle liposomes (Figure 3I). Mice depleted of monocytes also showed better performance in the treadmill and rotarod tasks as compared to nondepleted mice (Figure 3J,K), indicating the requirement of monocytes in surgery‐induced neuronal and learning deficits. In aged mice, depletion of monocytes by RB6‐8C5 or clodronate preserves spatial memory after surgery (Figure 3L–N and Figure S2B, Supporting Information). Collectively, these findings indicate that the impact of surgery on learning and learning‐dependent neuronal activity is mainly mediated by peripheral monocytes.

### 2.4 Monocytes Contribute to Increased IL‐1*β* Levels in the Cortex

**Figure 4:**
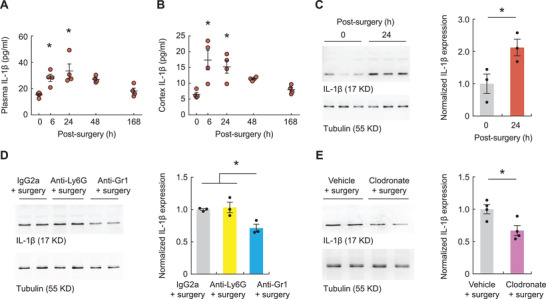
Monocytes contribute to increased cortical IL‐1*β* expression after surgery. A,B) Levels of IL‐1*β* in the A) plasma and B) cortex at various time points after surgery (*n* = 4 mice per group). C) Left, Western blotting of IL‐1*β* in the cortex 0 and 24 h after surgery. Right, quantification of data shown on the left. D) Left, Western blotting of IL‐1*β* in the cortex 24 h after surgery in mice that were injected with various antibodies. Right, quantification of data shown on the left. E) Left, Western blotting of IL‐1*β* in the cortex 24 h after surgery in mice that were injected with vehicle or clodronate liposomes. Right, quantification of data shown on the left (*n* = 4 mice per group). Throughout, each dot represents data from a single mouse. Summary data are presented as mean ± SEM. *p < 0.05; by one‐way ANOVA followed by Bonferroni's post hoc test (A, B, D), Student's *t* test (C and E). See also Table S1 in the Supporting Information.

The findings above indicate that blood monocytes are critical for surgery‐induced neuronal and learning deficits. To explore the molecular mechanisms underlying the mediating role of monocytes, we measured the protein levels of proinflammatory cytokines in the plasma and cortex at various time points after surgery using enzyme‐linked immunosorbent assays (ELISA). Surgery caused a transient increase in cytokine production in the circulation. Plasma levels of IL‐1*β*, TNF‐*α*, and IL‐6 were substantially increased at 6 h after surgery, relative to those in nonsurgery mice (**Figure** **4**A and Figure S7A,C, Supporting Information). Notably, we found a significant increase in IL‐1*β* levels in the cortex at 6–24 h after surgery (Figure 4B), whereas there were no lasting changes in TNF‐*α* and IL‐6 levels (Figure S7B,D, Supporting Information). Western blot analysis of the cortical tissue confirmed the increased IL‐1*β* levels at 24 h after surgery (Figure 4C).

To determine whether monocytes are required for surgery‐induced increase in IL‐1*β* levels in the cortex, we performed Western blot analysis in mice that were depleted of either monocytes or neutrophils (Figure 4D,E). At 24 h after surgery, we found that there were significantly less amounts of IL‐1*β* in the cortex of mice that were depleted of monocytes by either RB6‐8C5 or clodronate than in mice that had intact peripheral immune cells. By contrast, depletion of neutrophils by 1A8 had no significant effect on the IL‐1*β* level in the cortex, relative to the levels observed in nondepleted mice at 24 h after surgery (Figure 4D). These results suggest that monocytes are required for the increased brain IL‐1*β* levels after surgery.

### 2.5 Monocyte IL‐1*β* Mediates Surgery‐Induced Neuronal and Learning Deficits

**Figure 5:**
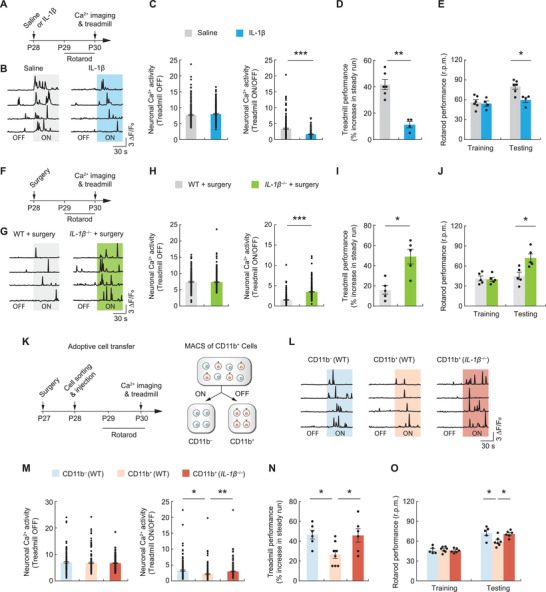
Administration of IL‐1*β* or IL‐1*β*‐producing CD11b cells sorted from surgery mice induces neuronal and behavioral deficits in naïve mice. A) Experimental timeline for IL‐1*β* administration, Ca^2+^ imaging, and behavioral testing. B) Ca^2+^ fluorescence traces of L5 pyramidal neurons in the primary motor cortex of mice that were injected with saline or exogenous IL‐1*β*. C) Left, neuronal Ca^2+^ activity during quiet resting. Right, fold change in neuronal Ca^2+^ activity during treadmill running versus rest (*n* = 5 mice per group). D) Treadmill and E) rotarod performance for the mice that were injected with saline or IL‐1*β*. F) Experimental timeline for surgery, Ca^2+^ imaging, and behavioral testing in WT and *IL‐1β* ^−/−^ mice. G–J) Similar to (B)–(E), but for WT and *IL‐1β* ^−/−^ mice with surgery (*n* = 5 mice per group). K) Experimental design. Leukocytes were collected from spleen tissues of WT or *IL‐1β* ^−/−^ donor mice at 24 h after surgery, and magnetically sorted based on their surface expression of CD11b. Nonsurgery mice were injected with 3 × 10^6^ CD11b^+^ or CD11b^−^ cells on P28. Following adoptive cell transfer, recipient mice were examined for motor learning and learning‐dependent neuronal activity. L) Ca^2+^ traces of L5 somas in the motor cortex of recipient mice that were transferred with CD11b^−^ or CD11b^+^ cells from WT surgery mice or CD11b^+^ cells from *IL‐1β* ^−/−^ surgery mice. M) Left, neuronal Ca^2+^ activity during quiet resting. Right, fold change in neuronal Ca^2+^ activity during treadmill running versus rest (4–5 mice per group). N) Performance in the treadmill task (*n* = 6–8 mice per group). O) Performance in the rotarod task (*n* = 5–7 mice per group). Summary data are presented as mean ± SEM. C,H,M) Each dot represents data from a single cell. D,E,I,J,N,O) Each dot represents data from a single mouse. *p < 0.05, **p < 0.01, ***p < 0.001; by Mann–Whitney test (C–E, H–J), one‐way ANOVA followed by Bonferroni's post hoc test (M–O). See also Table S1 in the Supporting Information.

Many lines of evidence indicate that IL‐1*β* activity can suppress neural plasticity and impair memory.^[^ ^39^, ^40^ ^]^ Given our findings that monocytes are required for surgery‐induced neuronal dysfunction and elevation of IL‐1*β* levels in the cortex, it is likely that the impact of surgery on learning and learning‐dependent neuronal activity is mediated by peripheral monocytes through IL‐1*β* signaling. To test this, we first examined neuronal activity in nonsurgery mice administered exogenous IL‐1*β* (**Figure** **5**A). 2 days after IL‐1*β* injection (50 µg kg^−1^, i.v.), we found that pyramidal neuronal activity was markedly decreased when mice run on the treadmill (Figure 5B,C). Consistent with the reduction of cortical neuronal activity, performance in the treadmill and rotarod tasks was significantly decreased after IL‐1*β* injection (Figure 5D,E), suggesting that elevated levels of IL‐1*β* may cause neuronal and learning deficits in mice. In support, *IL‐1β* ^−/−^ mice (which do not produce IL‐1*β*) were protected from surgery‐induced reduction in neuronal activity and motor learning impairment (Figure 5F–J).

We next investigated whether monocytes induce neuronal and learning impairments in an IL‐1*β*‐dependent manner. In this experiment, we collected leukocytes from the spleen tissue, a monocyte reservoir,^[^ ^41^ ^]^ of wild‐type (WT) and *IL‐1β* ^−/−^ mice at 24 h after surgery, and magnetically sorted them based on their surface expression of CD11b, a marker for myeloid cells including monocytes (Figure 5K). Nonsurgery mice were intravenously injected with 3 × 10^6^ sorted CD11b^−^ and CD11b^+^ cells. 2 days after adoptive cell transfer, we observed a substantial decrease of neuronal activity during motor learning in nonsurgery mice that received CD11b^+^ cells from WT mice compared to nonsurgery mice that received CD11b^−^ cells from WT mice (Figure 5L,M). Animals’ performance in the treadmill and rotarod tasks were markedly decreased (Figure 5N,O). Notably, there were no changes in neuronal activity and behavioral performance in nonsurgery mice that received CD11b^+^ cells collected from *IL‐1β* ^−/−^ mice (Figure 5L–O). These results indicate that administration of IL‐1*β*‐producing monocytes only is sufficient to induce neuronal dysfunction and learning impairment.

### 2.6 NLRP3 Inflammasome Activation Is Required for Increased IL‐1*β* Production and Neuronal and Learning Deficits

**Figure 6:**
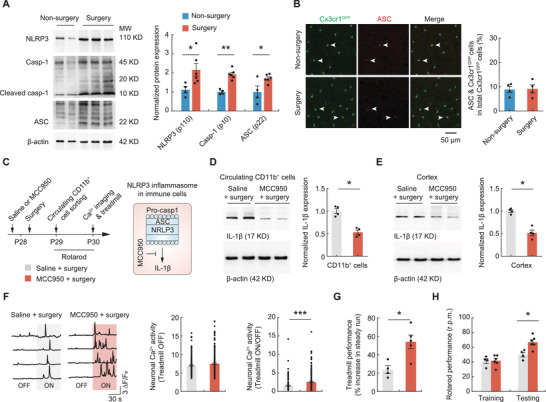
The NLRP3 inflammasome mediates monocyte IL‐1*β* production after surgery. A) Left, Western blotting of NLRP3 inflammasome components in leukocytes sorted from surgery (*n* = 6) or nonsurgery (*n* = 4) mice. Right, quantification of data shown on the left. B) Left, immunostaining of Cx3cr1^+^ microglia (green) and inflammasome adaptor protein ASC (red) in the cortex of surgery or nonsurgery mice. Right, quantification of the data shown on the left. C) Experimental timeline and schematic for inhibiting NLRP3 inflammasomes in mice before surgery. D) Western blotting and quantification of IL‐1*β* in circulating CD11b^+^ cells in mice that were injected with saline or MCC950 before surgery (*n* = 4 mice per group). E) Western blotting and quantification of IL‐1*β* in cortical tissues. F) Left, Ca^2+^ traces of L5 somas in the motor cortex during treadmill training in mice that were injected with saline or MCC950 before surgery. Middle, spontaneous neuronal Ca^2+^ activity. Right, fold change in neuronal Ca^2+^ activity during treadmill running compared to rest. G) Animals’ performance in the treadmill task. H) Performance in the rotarod task. Summary data are presented as mean ± SEM. F) Each dot represents data from a single neuron. A,B,D,E,G,H) Each dot indicates data from a single mouse. *p < 0.05, **p < 0.01, ***p < 0.001; by Mann–Whitney test. See also Table S1 in the Supporting Information.

The inflammasome signaling in monocytes serves as a molecular platform that senses the host‐derived damaged DNA and promotes the production of mature IL‐1*β*.^[^ ^42^, ^43^ ^]^ Previous studies using human blood samples have suggested that the innate immune system and NOD‐like receptor signaling pathways, such as IL‐1*β* and NLRC4, are activated within 24 h after major thoracoabdominal surgery (Figure S8, Supporting Information).^[^ ^12^ ^]^ To determine whether the inflammasome signaling is activated in mice after thoracic surgery, we sorted CD11b^+^ myeloid cells from blood and performed Western blotting of inflammasome components NLRP3, ASC, and caspase‐1. We found that the expression of NLRP3, ASC specks, and cleaved caspase‐1 was increased in circulating myeloid cells 24 h after surgery (**Figure** **6**A). In contrast, there were no signs of inflammasome activation or caspase‐mediated neuronal apoptosis or pyroptosis in the cortex, as shown by a similar number of ASC^+^ specks (Figure 6B) and NeuN^+^ cells (Figure S9, Supporting Information) in the cortex between surgery and nonsurgery mice.

To examine whether activation of the NLRP3 inflammasome in monocytes contributes to the increased IL‐1*β* levels and neuronal and learning deficits, we pharmacologically inhibited the function of NLRP3 inflammasomes by administering MCC950 (50 mg kg^−1^, i.p.), an inhibitor of NLRP3 inflammasomes (Figure 6C).^[^ ^44^ ^]^ Following surgery, MCC950‐treated mice had reduced amounts of IL‐1*β* both in circulating CD11b^+^ cells (Figure 6D) and in cortical tissues (Figure 6E) compared to saline‐treated mice. Moreover, surgery mice administered MCC950 showed a higher level of cortical neuronal activity during motor learning (Figure 6F) and better performance in the treadmill and rotarod tasks (Figure 6G,H). These data suggest that NLRP3 inflammasome activation increases IL‐1*β* production in monocytes, contributing to neuronal and learning impairment after surgery.

### 2.7 Monocytes Mediate Surgery‐Induced Synaptic Deficits

**Figure 7:**
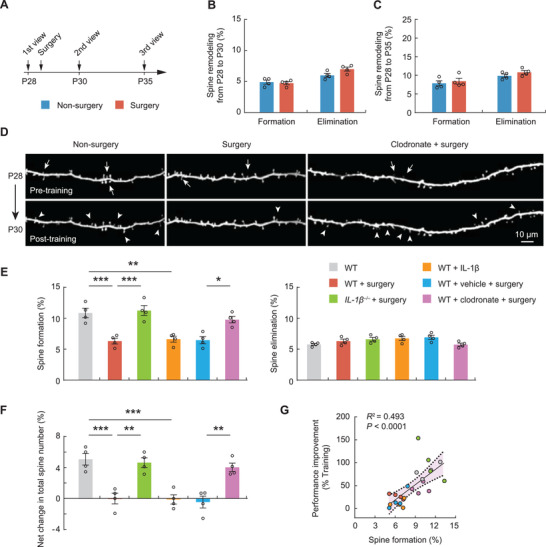
Surgery impairs learning‐dependent dendritic spine remodeling via monocyte IL‐1*β*. A) Schematic showing the timeline of surgery and in vivo two‐photon imaging. B,C) Summary quantification of dendritic spine elimination and formation over B) 2 days or C) 1 week in surgery or nonsurgery mice (*n* = 4 mice per group). D) Representative images of transcranial two‐photon imaging of dendritic spines on apical dendritic segments of L5 pyramidal neurons in the primary motor cortex of surgery or nonsurgery mice. Arrows and arrowheads indicate individual spines that were eliminated or newly formed, respectively, on the same dendritic segment after 2 days. E) Spine formation and elimination over 2 days in surgery or nonsurgery mice with various treatments (*n* = 4 mice per group). F) Net change in spine number at P30 for the mice in (E). G) Correlation between the number of learning‐induced new spines and the mouse's performance improvement (*R* ^2^ = 0.493; *p* < 0.0001). Throughout, each circle represents data from a single mouse. Summary data are presented as mean ± SEM. *p < 0.05, **p < 0.01, ***p < 0.001; by Mann–Whitney test (B, C), one‐way ANOVA followed by Bonferroni's post hoc test (E, F). See also Table S1 in the Supporting Information.

Finally, we investigated the synaptic effects of surgery by examining the dynamics of postsynaptic dendritic spines of L5 pyramidal neurons expressing yellow fluorescent protein (YFP) in the primary motor cortex of *Thy1*‐YFP mice. Comparing surgery and nonsurgery mice, we observed no difference in the rates of dendritic spine formation and elimination over 2 days and 1 week (**Figure** **7**A–C), indicating that surgery has no overt effects on the baseline spine dynamics in the mouse cortex.

Previous studies have shown that motor skill learning causes an increase in dendritic spine formation in the motor cortex, and that the extent of new spine formation correlates with the animals’ performance improvement after motor learning.^[^ ^45^, ^46^ ^]^ Given the detrimental effects of surgery on motor learning and learning‐dependent neuronal activity, we next investigated whether surgery affects learning‐dependent dendritic spine remodeling. To this end, we trained P28 mice in the rotarod task and imaged the same dendrites in the primary motor cortex before and after training (Figure 7D). 2 days after training, we found a significant decrease in motor learning‐induced spine formation (nonsurgery, 10.9 ± 1.5%, 656 spines from four mice; surgery, 6.3 ± 0.9%, 618 spines from four mice; *p* < 0.001) in surgery mice as compared with nonsurgery mice (Figure 7E). There was no difference in the rates of spine elimination. As a result, learning‐induced increase in total spine number was not observed in surgery mice (Figure 7F), indicating that major surgery causes a disruption of learning‐dependent dendritic spine remodeling.

Similar to what was observed in surgery mice, there was less new spine formation and net increase in total spine number in nonsurgery mice‐administered exogenous IL‐1*β* (Figure 7E,F). By contrast, surgery mice with IL‐1*β* knockout or monocyte depletion exhibited a higher increase in new spine formation and total spine number as compared to surgery mice with intact monocytes and IL‐1*β* production (vehicle liposomes + surgery, 6.5 ± 0.6%; clodronate liposomes + surgery, 9.8 ± 0.5%). We observed a significant correlation between the number of new spines formed after motor training and the animals’ performance improvement in the rotarod task (Figure 7G). Taken together, these data indicate that peripheral monocytes mediate cortical synaptic deficits after surgery.

## 3 Discussion

Despite the known involvement of innate immune system in PND, the underlying neuroimmune mechanisms are poorly understood. In this study, we showed that thoracic surgery in mice caused learning and memory impairment, which was accompanied by a reduction of cortical neuron activity and impairment of synaptic formation associated with motor learning. These neuronal and synaptic changes were prevented by in vivo ablation of peripheral monocytes or inhibition of NLRP3 inflammasomes, both of which attenuated the elevated IL‐1*β* signaling in response to surgery. Using adoptive cell transfer, we further showed that administration of IL‐1*β*‐producing monocytes was sufficient to mimic the adverse effects of surgery in nonsurgery mice. Together, our studies identified the neuronal underpinnings of PND and highlighted monocyte NLRP3‐IL‐1*β* signaling as a potential target for mitigating the cognitive effects of major surgery.

It is well known that major operation may cause behavioral and cognitive changes in the postoperative period, and subsets of patients with preoperative or intraoperative risk factors are particularly susceptible. In the present work, we found that both young and aged mice show impaired learning and memory after thoracic surgery. This could be due to 1) C57BL mice used in our study have more similar genetic background and thus exhibit more reproducible PND than human patients, and/or 2) thoracic surgery induces more robust deficits in learning and memory in mice that in human patients. Although the scale of incision is similar between thoracic surgery and abdominal surgery, thoracic surgery invades the thorax cavity and therefore may be more invasive. Indeed, a previous study in rats reported that postoperative neuroinflammation and cognitive dysfunction are more extended and severe after cardiac surgery versus abdominal surgery.^[^ ^47^ ^]^ To address these issues, future studies would be needed to investigate the susceptibility of PND in different mouse strains and in response to different surgical procedures.

Our studies showed that surgery, but not anesthesia, is the driving cause for PND and provided insights into its underlying pathophysiology. Thoracic surgery in mice causes pyramidal neuronal hypoactivity and synapse formation impairment in the cortex. Under physiological conditions, the firing activity of cortical neurons in rodents and primates is modified during motor learning to drive improved behavioral performance, while disrupting learning‐dependent neuronal activation renders difficulties to achieve better performance in behavioral tasks.^[^ ^31^, ^32^, ^48^, ^49^, ^50^ ^]^ In mice undergoing thoracic surgery, while there were no changes in spontaneous neuronal activity, we observed a marked decrease in cortical pyramidal neuron activity when mice learned to run on a treadmill. This decrease in running‐evoked neuronal activity lasted for at least 1 week, suggesting a lasting impact of major surgery on cortical circuits. Beyond neuronal hypoactivity, surgery caused an impairment in learning‐dependent synapse formation. Less new spines were formed in the motor cortex when mice were trained in a rotarod task during the postoperative period. Since learning‐dependent neuronal activation and dendritic spine formation are important for the animal's performance improvement after learning,^[^ ^45^, ^46^ ^]^ surgery mice had less performance improvement when they were retested in the treadmill or rotarod tasks the next day.

Our data highlight the role of monocytes in the neuropathology arising after major surgery. This was supported by the results of a series of in vivo experiments. First, the number of Ly6C^hi^ monocytes increased 24 h after surgery, while there were no changes in circulating neutrophils and B‐ and T‐lymphocytes, as well as microglia and brain macrophages. Second, surgery‐induced neuronal and behavioral alterations could be prevented by the depletion of peripheral monocytes using either anti‐Gr1^+^ mAb or clodronate liposomes. Third, adoptive transfer of IL‐1*β*‐producing myeloid cells from surgery mice mimicked the adverse effects of surgery in naïve mice. Finally, the NLRP3 inflammasome was activated in circulating myeloid cells but not microglia 24 h after surgery, and inhibition of NLRP3 inflammasomes attenuated neuronal and behavioral deficits after surgery. These results are consistent with previous findings in rodents that depletion of bone marrow‐derived myeloid cells prevents tibial surgery‐induced fear memory impairment in rodents.^[^ ^17^, ^18^ ^]^

Among all populations of monocytes, the Ly6C^hi^ subset is possibly involved in mediating PND. The Ly6C^hi^CCR2^+^ inflammatory monocytes (Gr1^+^ cells) in mice resemble CD14^+^CD16^−^ monocytes in human, while Ly6C^low^CCR2^−^ monocytes (Gr1^−^ cells) correspond to human CD14^dim^ CD16^+^ monocytes. Previous studies have shown that both CD14^+^ and CD14^dim^ monocytes can produce cytokines TNF‐*α*, IL‐1*β*, and type I interferon under various conditions such as infection,^[^ ^51^, ^52^ ^]^ inflammatory and autoimmune diseases,^[^ ^53^ ^]^ and cytokine‐release syndrome,^[^ ^54^ ^]^ though different signaling pathways may be involved. CD14^+^ human monocytes exhibit high phagocytic activity and produce cytokines and reactive oxygen species upon activation of TLR2/4 in response to a wide range of microbial cues, whereas CD14^dim^ monocytes have a weaker phagocytic ability and mainly produce cytokines in response to TLR7/8 ligands during viral infections.^[^ ^51^ ^]^ Given our findings that surgery increases the number of Ly6C^hi^ monocytes and that elimination of Gr1^+^ monocytes is neuroprotective, it is likely that Ly6C^hi^ (CD14^+^) monocytes are involved in mediating the cerebral effects associated with PND. These results in mice echo the finding of a previous human study that the recovery rates of surgical patients are inversely correlated with the number of CD14^+^ monocytes in blood.^[^ ^11^ ^]^

An important finding of the current study is the identification of NLRP3‐IL‐1*β* pathway in mediating PND and associated neuropathology. As potent proinflammatory cytokines, IL‐1*β* contribute to inflammatory conditions in many diseases including PND.^[^ ^13^ ^]^ The production and release of IL‐1*β* involves the activation of the NLRP3 inflammasome which detects both exogenous and host ligands and triggers an innate immune response.^[^ ^55^, ^56^, ^57^ ^]^ NLRP3 inflammasome activation leads to caspase‐1‐dependent cleavage of pro‐IL‐1*β* into their mature forms, allowing subsequent release. 1 day after thoracic surgery in mice, we detected a robust activation of NLRP3 inflammasomes in circulating myeloid cells, but not in cortical microglia. This inflammasome activation is critically involved in the activation of IL‐1*β* and changes of neuronal activity during the postoperative period, as pharmacological inhibition of NLRP3 inflammasomes prevented the elevation of IL‐1*β* signaling and restored learning‐dependent neuronal activity and behavioral improvement in mice. Furthermore, gene profiling of human blood leukocytes 24 h after thoracoabdominal surgery also suggests an upregulation of inflammasome‐related genes (Figure S8, Supporting Information).^[^ ^12^ ^]^ Thus, both animal and human data suggest that excessive activation of monocyte inflammasome pathway may play a role in PND.

The precise mechanisms by which monocyte IL‐1*β* impair neuronal and memory function remain unclear. Cytokines produced in the periphery can communicate with brain via multiple pathways (e.g., vagal afferents, humoral route).^[^ ^58^ ^]^ The fact that the concentration of IL‐1*β* is increased in both plasma and cortex within 6–24 h after surgery suggests that peripheral IL‐1*β* could reach the brain parenchyma by crossing the blood–brain barrier (BBB). Indeed, previous studies have shown that the mouse BBB is highly permeable after surgery.^[^ ^15^ ^]^ Once in the brain, IL‐1*β* can disrupt synaptic plasticity and long‐term potentiation by activating C‐Jun N‐terminal kinase and suppressing brain‐derived neurotrophic factor signal transduction.^[^ ^39^, ^59^, ^60^ ^]^ Aside from acting directly on neurons, IL‐1*β* can be sensed by astrocytes and endothelial cells where the expression of IL‐1 receptor has been revealed by RNA sequencing studies.^[^ ^61^ ^]^ It was reported that IL‐1*β* stimulation of endothelial cells led to the inflammatory gene expression in microglia,^[^ ^62^ ^]^ which can perpetuate an inflammatory response within the brain. Consistently, mice with global deficiency in IL‐1 receptor were partially protected from neuroinflammation and memory dysfunction after surgery.^[^ ^13^ ^]^

Although our data indicate that postoperative neuroinflammation and cognitive dysfunction can be initiated by monocyte IL‐1*β* signaling, eliminating monocyte function and inflammation in general may not be optimal in the perioperative period. It is known that monocytes and macrophages are critical for normal wound healing and tissue regeneration.^[^ ^63^ ^]^ During the inflammatory phase of healing, monocytes and macrophages release proinflammatory cytokines including IL‐1*β* to fight infection. Moderate levels of cytokines present in the wound also increase keratinocyte motility and proliferation. Thus, suppressing immune responses could negatively impact healing and increase patient vulnerability to infection. Future work will seek to address the challenge of disengaging periphery to the brain signaling to prevent neurological sequelae while minimizing off‐target effects.

In summary, our findings reveal changes of neuronal circuits associated with PND in mice. This surgery‐induced neuropathology is mediated by peripheral monocytes via NLRP3 inflammasome and IL‐1*β*‐dependent mechanisms. Targeting monocyte IL‐1*β* signaling to the brain represents a possible strategy to prevent cognitive decline after major surgery.

## 4 Experimental Section

### Animals

*Thy1.2*‐GCaMP6s line 1 mice were obtained from the laboratory of Wen‐Biao Gan at New York University.^[^ ^64^ ^]^ *Cx3cr1* ^GFP^ mice (Stock 005582), *Il‐1β* ^−/−^ mice (Stock 034447), and *Thy1*‐YFP‐H mice (Stock 003782) were purchased from the Jackson Laboratory. Mice were group‐housed in temperature‐controlled rooms on a 12 h light–dark cycle and were randomly assigned to different treatment groups. Both male and female mice were used. All mice were maintained in the animal facility at Columbia University Medical Center and handled in accordance with the NIH guidelines for animal care and use. All animal procedures (Protocol #: AC‐AABN7553) were approved by Institutional Animal Care and Use Committee at Columbia University.

### Mouse Model of Thoracic Surgery

The study targeted the delayed neurocognitive recovery of PND because the effects of surgery on the behavioral changes were measured within 1 week.^[^ ^2^ ^]^ Thoracic surgery was performed using sterile techniques as previously described with minor modifications.^[^ ^47^ ^]^ Specifically, mice were anesthetized with 1.5% isoflurane in air. After shaving the hair on the left thoracic cage, a 1.5 cm long incision on the skin and muscles was made over the 4th and 5th intercostal space to create a surgical window. The window was maintained for 3 min to mimic the surgical exposure. Organs inside the chest wall were not touched. The incision was then sutured layer by layer with 6‐0 absorbable and nylon sutures. The procedure for each animal lasted about 15 min. Throughout the procedure, the animals’ body temperature was maintained at ≈37 °C and respiratory rate was around 80 breaths per min during anesthesia. The animal was continuously monitored until recovered from anesthesia, and then returned to its home cage with food and water available ad libitum. Buprenorphine (1.5 mg kg^−1^, i.p.) was administered daily for 2 days to provide post‐operative analgesia. 1 day post‐surgery, mice in the surgery group showed normal locomotor function with no signs of untreated pain or distress (Figure S1, Supporting Information). The mice in the nonsurgery control group were placed in the home cage with room air for 15 min. The mice in the anesthesia only group received 1.5% isoflurane in air for 15 min. The experimenters were blinded to treatment groups during behavioral, Ca^2+^ imaging, and histological assessments.

### Behavioral Assays

All behavioral experiments were performed in a double‐blind fashion.

*Grooming*: The mouse was placed in an acrylic cage (5 cm × 9 cm × 5 cm). During a 10 min period, the number of self‐grooming episodes was counted manually.

*Open field test*: The mouse was allowed to freely explore an open field arena (40 cm × 40 cm) in a 5 min test session. The ANY‐maze software (Stoelting, Wood Dale, IL) was used to perform video tracking and analyze the total distance traveled, average speed, and time spent in the center of the arena.

*Rotarod*: An EZ Rod system with four test chambers (Accuscan Instruments, Columbus, OH, USA) was used in this study. Mice were individually placed on the motorized rod (30 mm in diameter) in each chamber. The rotation speed of the rod was linearly increased from 0 to 100 r.p.m. over the course of 3 min. The time latency and rotation speed were recorded by the software when the animal was unable to keep up with the increasing speed and fell. Rotarod training was performed in three sessions (20 trials each session) on the training day and testing was performed the next day. Performance was measured as the average speed that animals achieved during the training/testing session.

*Treadmill*: A custom‐built treadmill was used to provide motor training experience to mice head‐fixed on the stage of the two‐photon microscope. Specifically, mice were subjected to one treadmill training session which was consisted of 20 running trials. Following the start of a running trial, the treadmill was turned on and the speed of belt gradually increased from 0 to 8 cm s^−1^ within ≈3 s and maintained constant afterward. Upon completion of a 60 s running trial, the treadmill was turned off and mice were allowed to rest on the stationary treadmill for at least 3 min until the next trial began. To record the animal's gait pattern for behavioral analysis, a piece of white paper was laid over the treadmill belt and the animal run on it with paws coated with ink. The footprint was analyzed manually as previously described.^[^ ^30^ ^]^ Pre‐training performance was measured from footprints collected during the first five running trials in the training session. Post‐training performance was measured from footprints collected during the last five running trials.

*T maze*: T maze is a standard test for assessing the animal's spatial memory, based on the mouse's natural tendency to explore the novel location. The test was conducted in a T maze chamber (Stoelting, Wood Dale, IL) which was consisted of three arms of equal length. During the training session, mice were placed in the designated start arm and allowed to access two of three arms for 2 min. A removable door was used to prevent the mice from accessing the third arm. During the testing session, mice were returned to the start location in the T maze and allowed to explore all three arms for 2 min. An infrared digital camera was mounted above the maze and interfaced with ANY‐Maze software to automatically record the time spent and the number of entries in each arm. Novel zone preference was calculated as the time spent in the novel arm divided by the time spent in both novel and familiar arms.

### In Vivo Ca^2+^ Imaging and Data Analysis

The surgical procedure for in vivo Ca^2+^ imaging in awake head‐restrained mice was described previously.^[^ ^30^ ^]^ In short, mice were anesthetized with 100 mg kg^−1^ ketamine and 15 mg kg^−1^ xylazine. After a head frame was attached to the mouse's skull surface using glue and dental acrylic, a cranial window was created over the forelimb region of the primary motor cortex based on stereotactic coordinates (1.3 mm anterior to the bregma and 1.2 mm lateral from the midline). The completed cranial window was covered with silicon elastomer and mice were given at least 24 h to recover. Later, mice were habituated to the imaging platform three times (10 min each) to minimize the potential stress associated with head fixation and imaging. Right before imaging, the silicon elastomer was removed and the animal was head‐fixed and placed on the stage of a Scientifica two‐photon microscope equipped with a Ti:Sapphire laser (Vision S, Coherent) tuned to 920 nm. Time‐lapse images were acquired at a frame rate of 1.07 Hz and an image size of 512 × 512 pixels, through a 20× objective (1.0 N.A.) immersed in artificial cerebrospinal fluid. Image acquisition was performed using ScanImage software (Vidrio Technologies). Because mice tended to struggle when they first run on the treadmill, Ca^2+^ recording was collected during the fifth running trial.

Image analysis was performed using NIH ImageJ software as previously described.^[^ ^65^ ^]^ All raw images were registered and motion corrected using TurboReg plugin for ImageJ. Regions‐of‐interests (ROIs) corresponding to visually identifiable tuft/trunk dendrites and somas were selected manually. For each ROI, after the subtraction of background fluorescence, all the pixels within the ROI were averaged to obtain a time‐series fluorescence trace which was expressed as Δ*F*/*F*_0_ = (*F* − *F*_0_)/*F*_0_, where *F*_0_ is the baseline fluorescence averaged over a 6 s period corresponding to the lowest fluorescence signal during the recording period. The Ca^2+^ activity was quantified as the average of Δ*F*/*F*_0_ over a 1 min recording collected during treadmill off or on. Running evoked neuronal responses were calculated as Ca^2+^ activity during treadmill on normalized by Ca^2+^ activity during treadmill off.

### Immunohistochemistry and Image Analysis

Mice were deeply anesthetized and perfused with 4% paraformaldehyde. Whole brain tissues were dehydrated with 30% sucrose for 48 h and sectioned into 60 µm coronal slices using a Leica VT 1000 S vibratome. The brain slices were rinsed twice in phosphate buffered saline (PBS), blocked with goat serum at room temperature for 1 h, and then incubated at 4 °C for 24 h with the primary antibodies: rabbit anti‐Iba‐1 (Wako, 019–19741, 1:500), rabbit anti‐ASC (CST, 67824, 1:500), rabbit anti‐NeuN (Abcam, ab177487, 1:500), and Alexa Fluor 647 anti‐mouse CD206 (BioLegend, 141712, 1:500). Brain slices were washed twice in PBS and incubated for 1 h with the secondary antibody, goat anti‐rabbit Alexa Fluor 488. Sections were washed twice and mounted by 4′,6‐diamidino‐2‐phenylindole. Images were captured using a confocal microscope (Nikon, Japan) and analyzed using ImageJ software. All images meant for comparison were uniformly processed.

Microglia density and morphology were analyzed from maximum intensity projection images created from a 30 µm thick z stack of optical sections taken at 2 µm increments of the primary motor cortex. Microglia density was determined by counting all Iba1^+^ cells within a 318.7 µm × 318.7 µm field of view in the cortex. One brain section was analyzed per animal. For each optical image, 6–10 Iba1^+^ cells were randomly selected for further analysis of soma size, soma mean fluorescent intensity, and major branch number.

### Flow Cytometry

Peripheral blood mononuclear cells (PBMCs) were analyzed using flow cytometry as previously described.^[^ ^66^ ^]^ Mice were deeply anesthetized, and blood was collected via cardiac puncture. Whole blood sample was incubated with red blood cell lysis buffer at room temperature for 10 min. Following a centrifugation at 400*g*, 4 °C, for 5 min, PBMCs were harvested, washed twice with PBS, and centrifuged at 400*g*, 4 °C for another 5 min. Cells were resuspended in 200 µL flow cytometry staining buffer. Nonspecific binding to Fc receptors was blocked by incubation with a CD16/32‐specific antibody (Biolegend, 101302) at 4 °C for 15 min. Cells were washed and stained with following antibodies: anti‐mouse CD45 brilliant violet 711 (Biolegend, 103147), anti‐mouse CD11b PE (Biolegend, 103147), anti‐human CD3 Brilliant violet 786 (Biolegend, BDB563918), anti‐mouse B220 PE‐Cyanine5.5 (Biolegend, 35045282), anti‐mouse Ly6C FITC (Biolegend, 128006), anti‐mouse mouse Ly6G Pacific blue (Biolegend, 127612), and anti‐mouse F4/80 PE‐Cyanine 7 (Biolegend, 123114). Flow cytometry was performed on an LSRII (BD) and analyzed with FlowJo v 10.4.

### Depletion of Monocytes and/or Neutrophils

To deplete monocytes and neutrophils in vivo, mice were intravenously (i.v.) injected 300 µg anti‐Gr‐1 mAb RB6‐8C5 (Bio X cell, BE0075) 24 h before surgery. To selectively deplete neutrophils, 300 µg anti‐Ly6G mAb 1A8 (Bio X cell, BE0075‐1) were injected i.v. into the mice. Control animals were injected 300 µg IgG2a isotype control (Bio X cell, BP0089). In a separate set of experiments to selectively deplete monocytes, mice were administered i.v. 250 µg clodronate liposomes (Encapsula NanoSciences LLC, NC0302518) 24 h before surgery. Control mice received vehicle liposome injection.

### Magnetic Activated Cell Sorting

CD11b^+^ cells were harvested from blood and spleen tissues by magnetic cell sorting (MACS). Leukocytes were isolated from blood after centrifugation in a lymphocyte separation medium (Corning, 25072CI) at 300*g*, 4 °C for 30 min. To isolate leukocytes from spleen, spleen tissue was dissected and smashed in MACS buffer. The cell suspension was passed through a 70 µm cell strainer, centrifuged at 300*g*, 4 °C for 10 min. Blood and spleen leukocytes were resuspended in MACS buffer and incubated with CD11b microbeads (Miltenyibiotec, 130‐126‐725) at 4 °C for 15 min. Magnetic separation was performed in MACS separators. Cell viability was examined by a trypan blue exclusion assay and cell number was counted using a hemocytometer. Sorted CD11b^−^ and CD11b^+^ cells were intravenously transferred into the recipient mice or frozen storage at −80 °C for Western blot analysis.

### Western Blotting and ELISA

Mice were euthanized and cortical lysates were extracted using the cocktail of protease and phosphatase inhibitor (Thermo Scientific, 78442). Lysates were stored at −80 °C when not used immediately. Protein quantification was performed using Pierce BCA protein assay kit (Thermo Scientific, 23227). Protein samples (10 µg) were added to each lane for sodium dodecyl sulfate‐polyacrylamide gel electrophoresis separation and polyvinylidene fluoride‐membrane transferring. The membranes were washed twice in PBST, blocked with 5% bovine serum albumin, and then incubated at 4 °C overnight with primary antibodies: rabbit anti‐IL‐1*β* (CST, 83186, 1:1000), rabbit anti‐NLRP3 (CST, 15101, 1:1000), rabbit anti‐Caspase‐1 (CST, 3866, 1:1000), anti‐Tubulin (CST, 5568, 1:1000), and anti‐ *β*‐Actin (CST, 3700, 1:1000). The membranes were washed in PBST and incubated for 2 h with secondary antibodies: HRP‐linked anti‐rabbit IgG (CST, 7074, 1:2000) or anti‐mouse IgG (CST, 7076, 1:2000). Protein bands were visualized using an imaging system (ImageQuant LAS‐4000). Protein expression difference was measured using Image J software.

For ELISA, whole blood was collected and centrifuged at 2000*g*, 4 °C, for 10 min to harvest the plasma fraction. Cortex and plasma samples were then measured for the amounts of TNF‐*α*, IL‐6, and IL‐1*β* using the TNF‐*α* Mouse ELISA kit (Invitrogen, BMS6073), IL‐6 Mouse ELISA kit (Invitrogen, KMC0061), and IL‐1*β* Mouse ELISA kit (Invitrogen, BMS6002TWO) according to the manufacturer's instructions.

### In Vivo Imaging of Dendritic Spines and Data Analysis

The surgical procedure for transcranial two‐photon imaging of dendritic spines was described previously.^[^ ^67^ ^]^ In brief, the animal was anesthetized, and a custom‐made head plate was glued to the skull with the central opening over the primary motor cortex. A thinned‐skull window (200 µm in diameter, 20 µm in thickness) was created by using a dental drill and a microsurgical blade. The anesthetized mice were then placed under a Scientifica two‐photon microscope with the laser tuned to the optimal excitation wavelength for YFP (920 nm). Using a 25× water‐immersion objective (1.05 N.A.), five to eight stacks of image planes within a depth of 100 µm from the pial surface were collected at a digital zoom of 1 to 4×. After image acquisition, the head plate was gently detached from the skull, and the scalp was sutured with 6‐0 thread. The animals were returned to their home cages until the next view.

Data analysis was performed using NIH ImageJ software. The same dendritic segments were identified from 3D image stacks taken at both time points. The number and location of dendritic protrusions were identified in each view. Filopodia were identified as long, thin structures without enlarged heads, and the rest of the protrusions were classified as spines. Spines were considered the same between two views based on their spatial relationship to adjacent spines and other landmarks. Spines in the second view were considered different if they were more than 0.7 µm away from their expected positions based on the first view. The formation or elimination rates of spines were measured as the number of spines formed or eliminated divided by the number of spines existing in the first view.

### Statistical Analysis

Prism 7.05 software (GraphPad) was used to conduct the statistical analysis. Summary data were presented as mean ± SEM. Tests for differences between two groups were performed using the unpaired two‐tailed Student's *t* test or Mann–Whitney test. Multiple‐group comparison was performed with one‐way or two‐way ANOVA followed by Bonferroni's post hoc test as specified in the figure legends. No data points were excluded from the statistical analysis. Significance was set at *p* < 0.05.

## Conflict of Interest

The authors declare no conflict of interest.

## Author Contributions

K.C. and G.Y. designed research studies; K.C. performed in vivo imaging, cell sorting, and behavioral experiment; K.C. performed biochemical experiments with assistance of Q.H.; K.C. analyzed data with input from G.Y. and Z.X.; K.C., G.Y., Z.X. wrote and revised the manuscript.

## Supporting information

Supporting information

## Data Availability

The data that support the findings of this study are available from the corresponding author upon reasonable request.
