# Supplementary material for: Monocyte NLRP3‐IL‐1β Hyperactivation Mediates Neuronal and Synaptic Dysfunction in Perioperative Neurocognitive Disorder
Source: Adv Sci (Weinh). 2022 Mar 28;9(16):2104106. doi: 10.1002/advs.202104106 (PMC9165480; doi:10.1002/advs.202104106)
Supplement: Supplementary file 1 — Supporting information [file ADVS-9-2104106-s001.pdf]

Supporting Information

Monocyte NLRP3-IL-1β hyperactivation mediates neuronal and synaptic dysfunction in perioperative neurocognitive disorder

Kai Chen, Qiuping Hu, Zhongcong Xie, Guang Yang

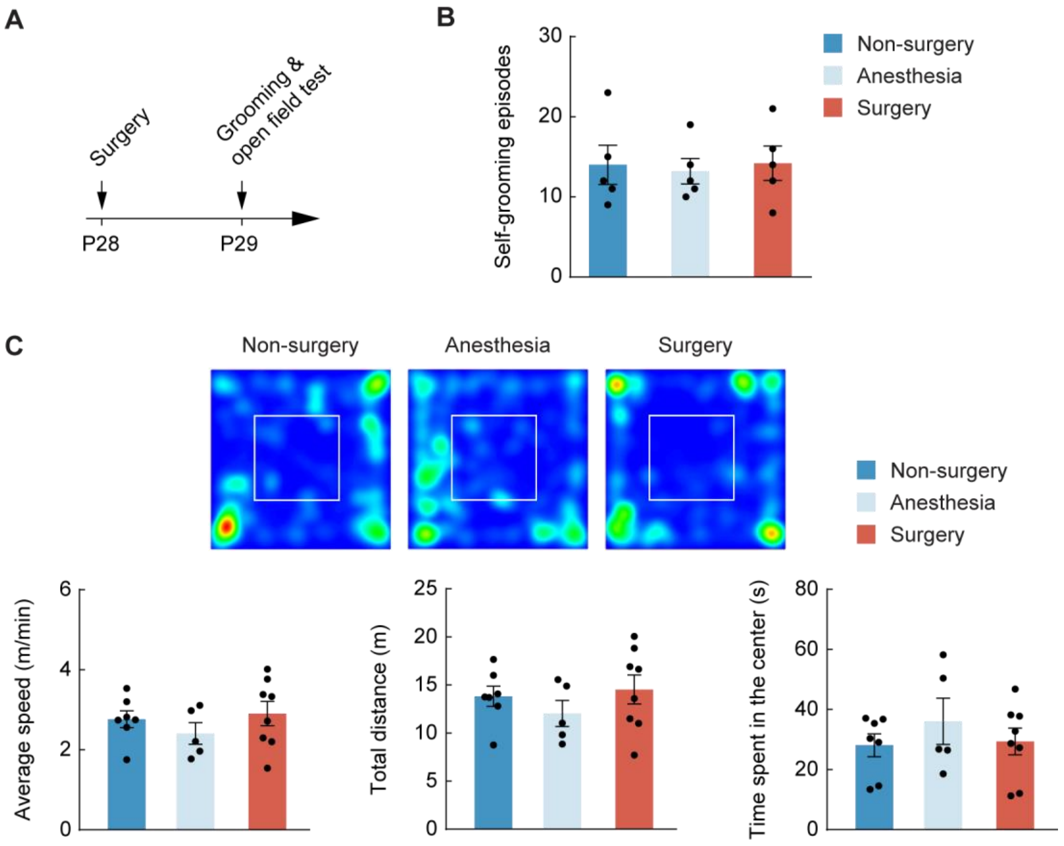

Figure S1. Surgery has no effect on grooming and locomotor activity in mice.
(**A**) Experimental timeline. (**B**) Mice showed normal self-grooming activity 1 day after anesthesia and surgery (*n* = 5 mice per group). (**C**) Top: representative heatmap showing the accumulative time mice spent in each area of the open field arena. Bottom: measures of the average speed, total distance traveled, and time spent in the center of the open field arena (*n* = 5–8 mice per group). Each dots represent data from a single mouse. Summary data are presented as mean ± s.e.m. One-way ANOVA. See also **Table S1**.

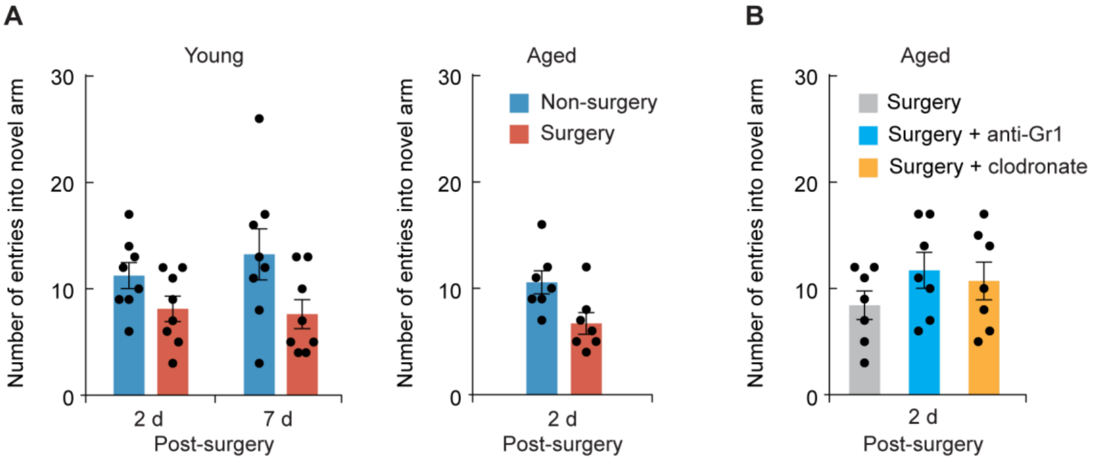

Figure S2. Analysis of the animal's exploratory behavior in the T maze test.
(**A**) During a 2-min test session, number of entries into the novel arm of T maze was counted. Surgery mice, compared to age-matched non-surgery controls, showed a trend of reduced entries into the novel arm, although it was not statistically significant (*n* = 7–8 mice per group). (**B**) Number of entries into the novel arm of T maze for aged mice that were injected with anti-Gr1 antibody or clodronate liposomes before surgery (*n* = 7 mice per group). Surgery mice depleted of monocytes showed a trend of increased entries into the novel arm, although it was not statistically significant. Each dots represent data from a single mouse. Summary data are presented as mean ± s.e.m. Mann–Whitney test or one-way ANOVA followed by Bonferroni's *post hoc* test was used. See also **Table S1**.

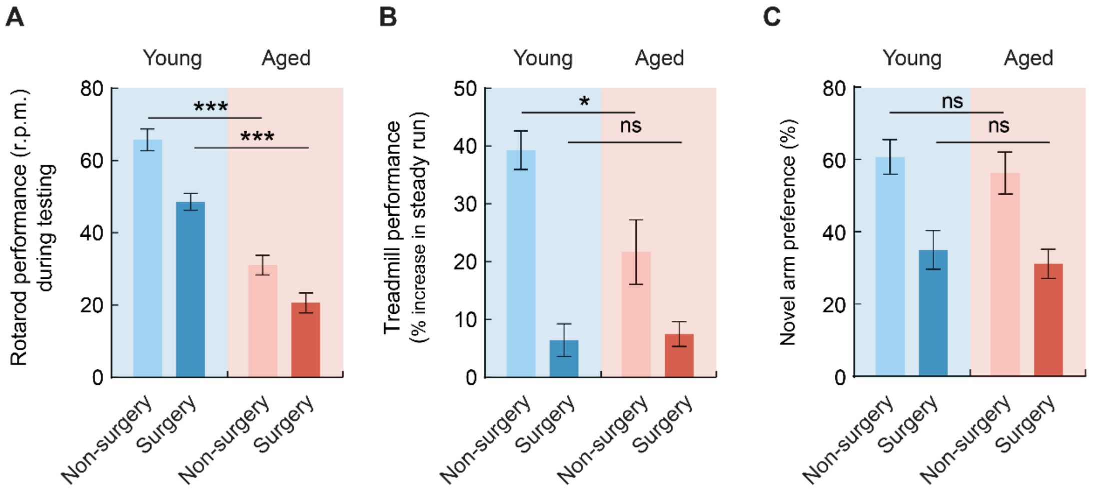

Figure S3. Effects of age and surgery on learning and memory in mice.
(**A**) Rotarod performance for young (1 month) and aged (> 18 months) mice, with or without surgery. In both non-surgery and surgery groups, young mice had better rotarod performance than aged mice. Surgery factor, F (1, 27) = 24.9, P < 0.0001; age factor, F (1, 27) = 128.2, P < 0.0001. Interaction, F (1, 27) = 1.48, P = 0.2342, two-way ANOVA. (**B**) Treadmill performance for young and aged mice, with or without surgery. In non-surgery group, young mice showed better performance in the treadmill task than aged mice. In surgery group, there was no difference in treadmill performance between young and aged mice. Surgery factor, F (1, 22) = 41.85, P < 0.0001; Age factor, F (1, 22) = 5.182, P = 0.0329. Interaction, F (1, 22) = 6.611, P = 0.0174, two-way ANOVA. (**C**) Performance in the T maze test for young and aged mice, with or without surgery. In both non-surgery and surgery groups, age had no significant effect on the animals' performance in the T maze test. Surgery factor, F (1, 26) = 25.1, P < 0.0001; Age factor, F (1, 26) = 0.6747, P = 0.4189; Interaction, F (1, 26) = 0.003147, P = 0.9557, two-way ANOVA. Summary data are presented as mean ± s.e.m. ns, not significant, *P < 0.05, ***P < 0.001; by two-way ANOVA followed by Bonferroni's *post hoc* test.

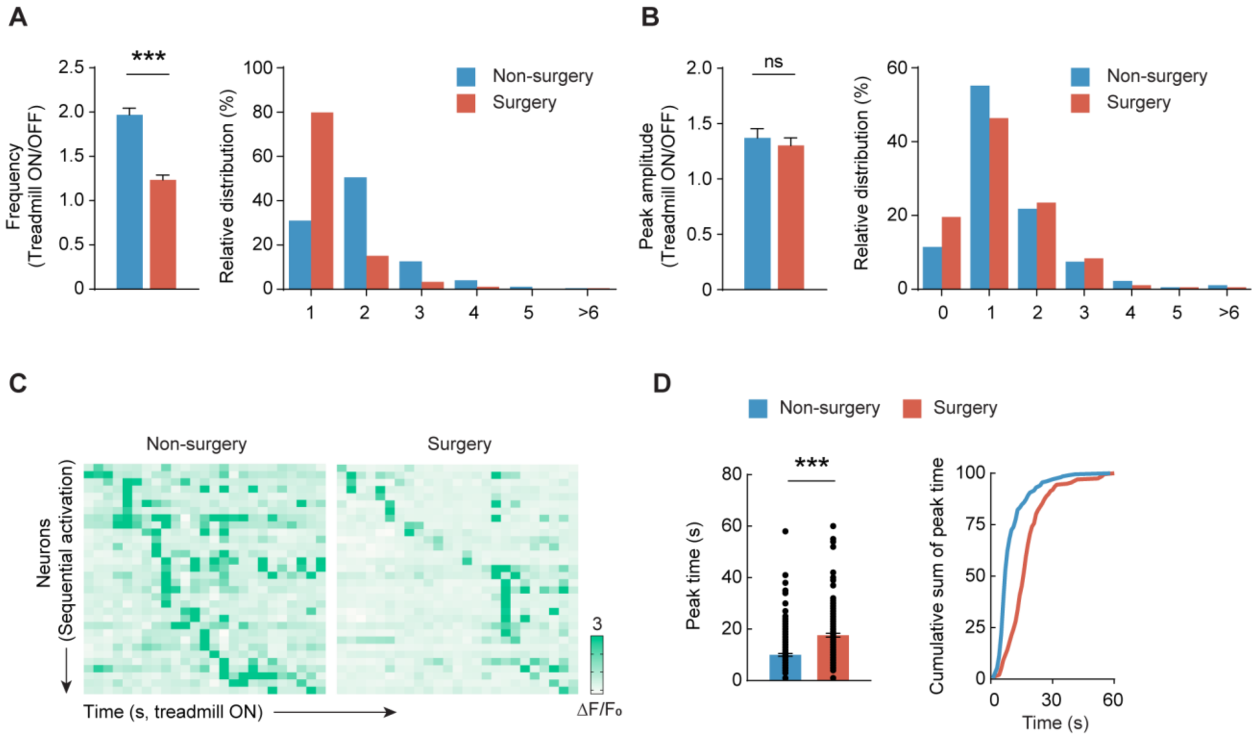

Figure S4. Surgery impairs cortical neuronal activity during treadmill running.
(**A, B**) Fold change in pyramidal neuron somatic Ca^{2+} frequency (**A**) and peak amplitude (**B**) during treadmill running versus rest (5 mice per group). Compared to non-surgery controls, surgery mice showed lower Ca^{2+} frequency during treadmill running. (**C**) Heatmap showing Ca^{2+} activity in individual pyramidal neurons of the motor cortex when the mouse was running on a treadmill. (**D**) Time for individual neurons to reach their peak activity during treadmill running (5 mice per group). Surgery mice showed a delayed neuronal activation compared to non-surgery mice. Each dots represent data from a single cell. Summary data are presented as mean ± s.e.m. ***$P < 0.001$ by Mann–Whitney test. See also **Table S1**.

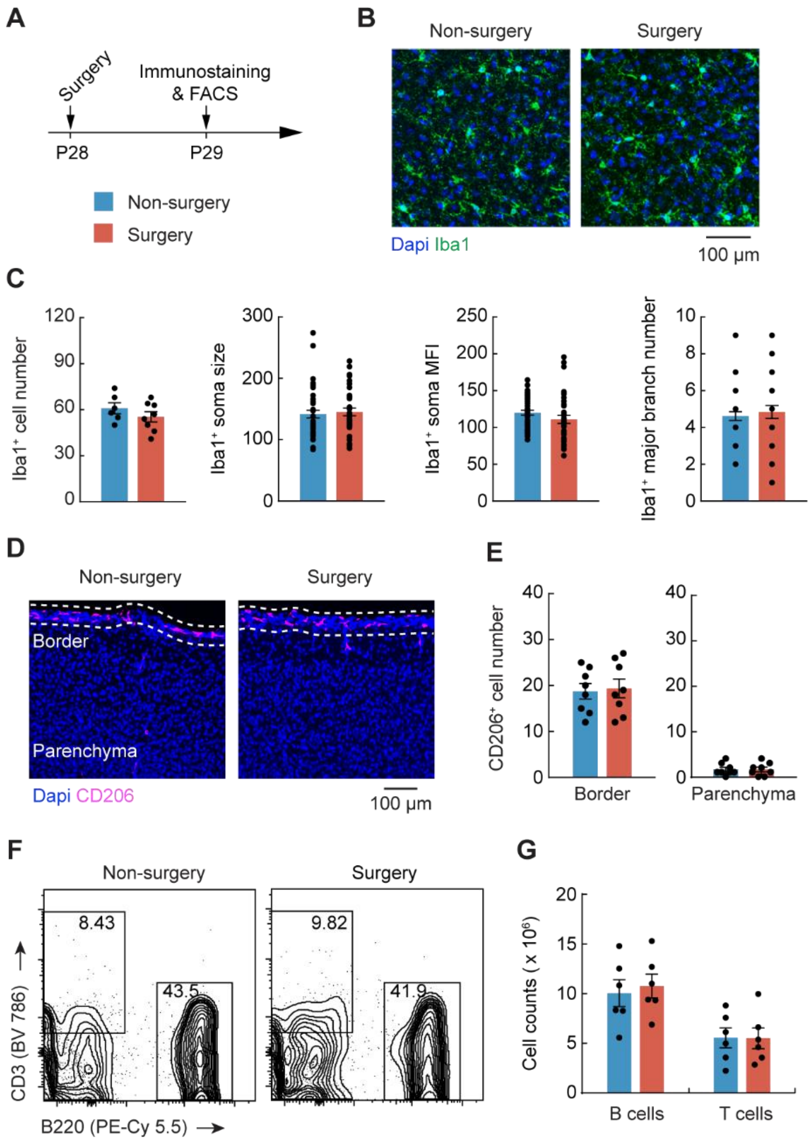

Figure S5. Surgery has no effect on brain macrophages and circulating lymphocytes at 24 h. (**A**) Experimental timeline for surgery, immunostaining, and fluorescence-activated cell sorting (FACS) analysis. (**B**) Representative coronal sections of the mouse cortex stained with the microglia marker Iba1 (green) and nuclei marker Dapi (blue). Scale bar, 100 μm. (**C**) Quantification of Iba1$^+$ cell density, soma size, soma mean fluorescent intensity (MFI) and major branch number (non-surgery, *n* = 6 mice; surgery, *n* = 8 mice; one section quantified per animal, 6–10 cells quantified per section). (**D**) Representative coronal sections of the mouse cortex stained with the macrophage marker CD206 (magenta) and nuclei marker Dapi (blue). (**E**) Quantification of CD206$^+$ cells in the border and parenchyma of the cortex in mice with or without surgery (*n* = 8 mice per group). (**F**) Representative FACS analysis showing the percentages of B220$^+$ B cells and CD3$^+$ T cells in the blood of surgery or non-surgery mice at 24 h. Rectangular boxes indicate B220$^+$ B lymphocyte and CD3$^+$ T lymphocyte population in the

blood. Gate: singlets, CD11b^{-}CD45^{hi}. (**G**) Quantification of the data shown in **F**. Summary data are presented as mean ± s.e.m. Mann–Whitney test was used. See also **Table S1**.

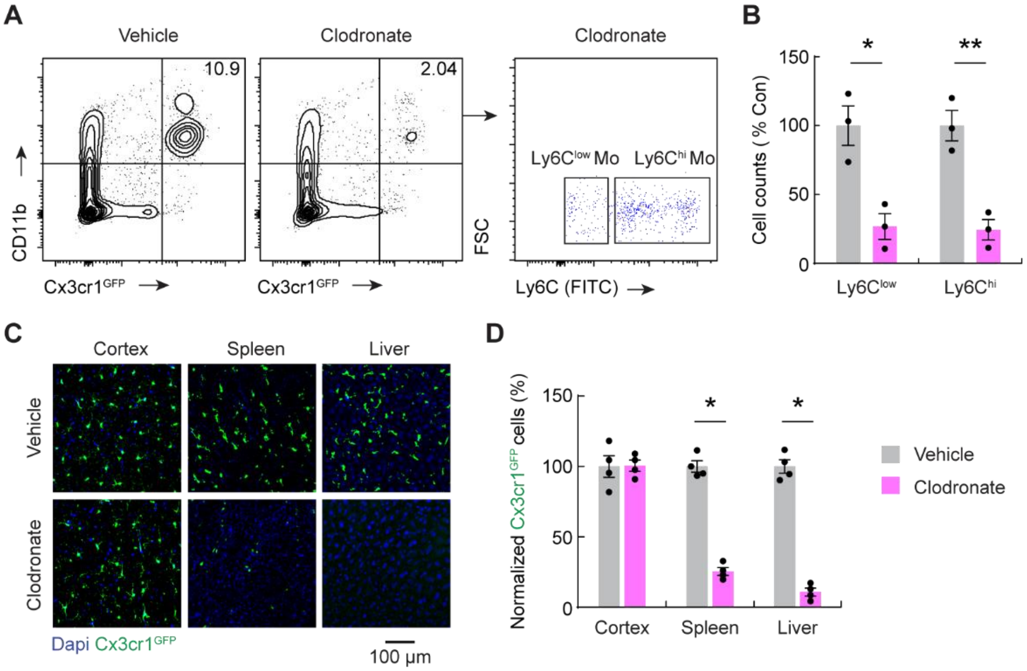

Figure S6. Administration of clodronate liposomes depletes circulating monocytes but has no effect on brain-resident microglia.

(**A**) Representative flow cytometry analysis showing the percentages of CD11b^{+}Cx3cr1^{GFP} cells at 24 h after surgery in the blood of mice that were injected with vehicle or clodronate liposomes. (**B**) Fold changes of Ly6C^{low} and Ly6C^{hi} monocyte counts in the blood of mice that were injected with clodronate versus vehicle liposomes (n = 3 mice per group). (**C**) Representative sections of the motor cortex, spleen, and liver from Cx3cr1$^{GFP/+}$ mice that were injected with vehicle or clodronate liposomes. (**D**) Fold changes in Cx3cr1^{+} cell number in the cortex, spleen and liver of mice that were injected with clodronate versus vehicle liposomes (n = 4 mice per group). Each dots represent data from a single mouse. Summary data are presented as mean ± s.e.m. *P < 0.05, **P < 0.01; by Student's t test (**B**) or Mann–Whitney test (**D**). See also **Table S1**.

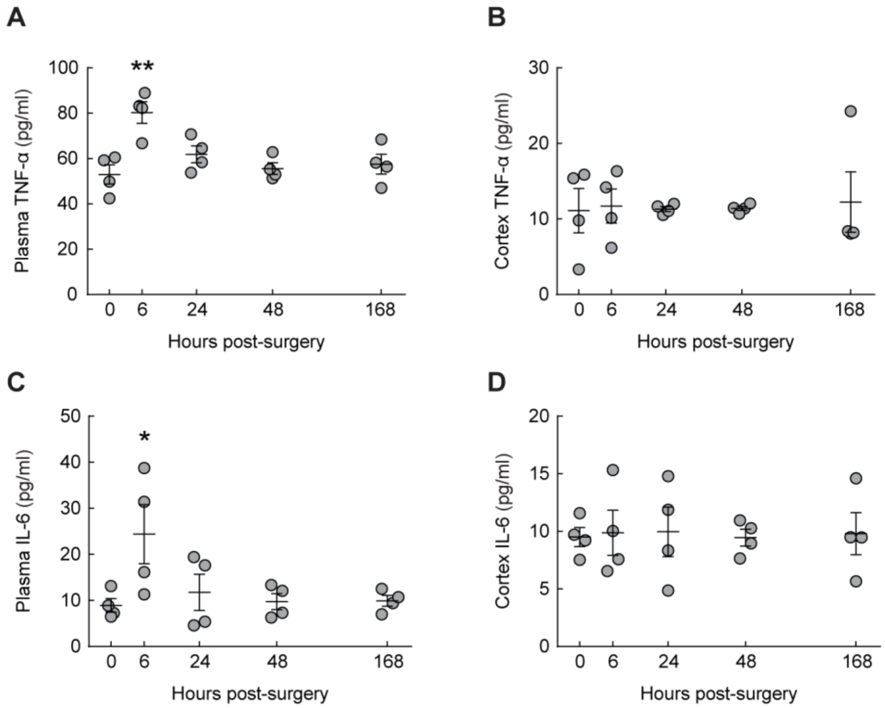

Figure S7. Protein levels of TNF-α and IL-6 in the plasma and cortex.
(**A, B**) Levels of TNF-α in the plasma (**A**) and cortex (**B**) at various time points after surgery (n = 4 mice per group). (**C, D**) Levels of IL-6 in the plasma (**C**) and cortex (**D**) at various time points after surgery (n = 4 mice per group). Each dots represent data from a single mouse. Summary data are presented as mean ± s.e.m. $^{*}P < 0.05$, $^{**}P < 0.01$; by one-way ANOVA followed by Bonferroni's *post hoc* test. See also **Table S1**.

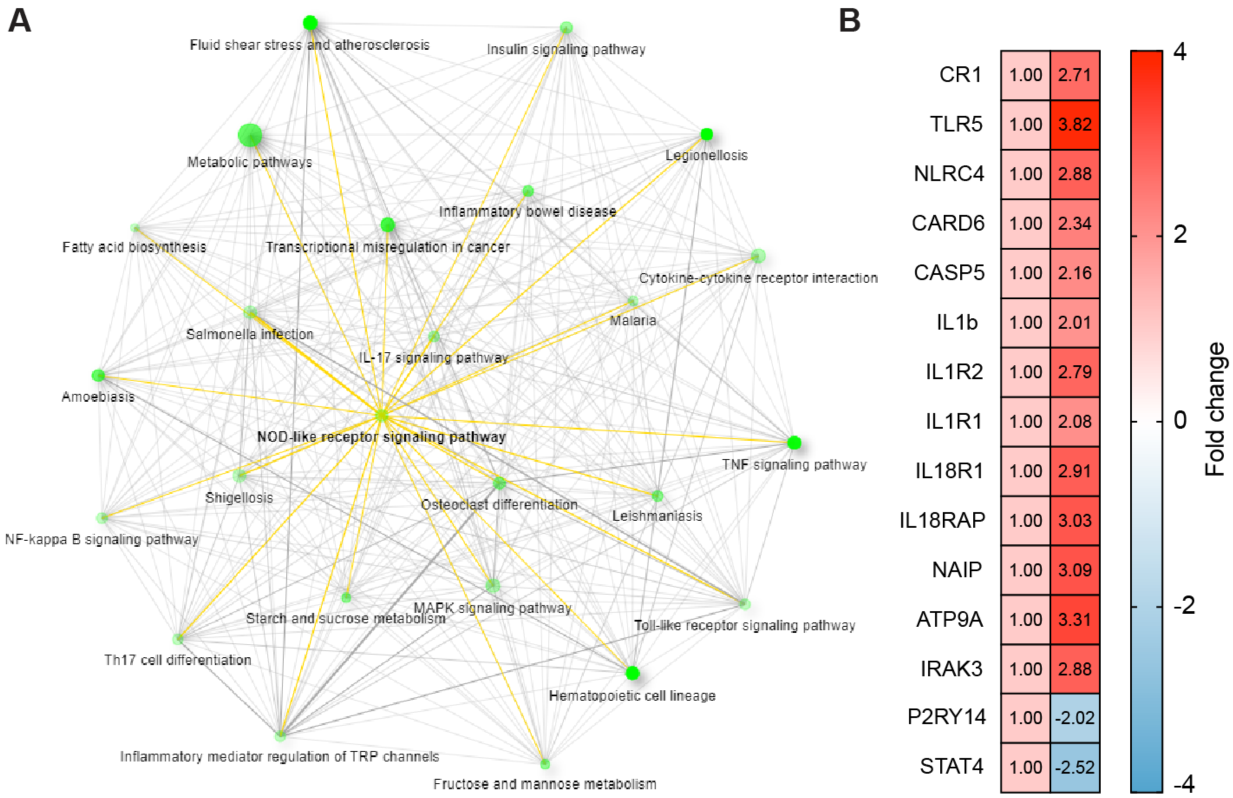

Figure S8. Surgery activates inflammasome signaling pathways in human patients.
(**A**) Gene expression profiling of circulating leukocytes in patients undergone thoracoabdominal surgery to identify enriched signaling pathways at 24 h. Signal pathway enrichment analysis by ShinyGO v0.66 shows that NOD-like receptor pathways are predominantly activated and crosstalk with other innate immune signaling pathways, such as Toll-like receptors and cytokine receptors. (**B**) Changes of inflammasome-related genes in circulating leukocytes of patients at 24 h after thoracoabdominal surgery.

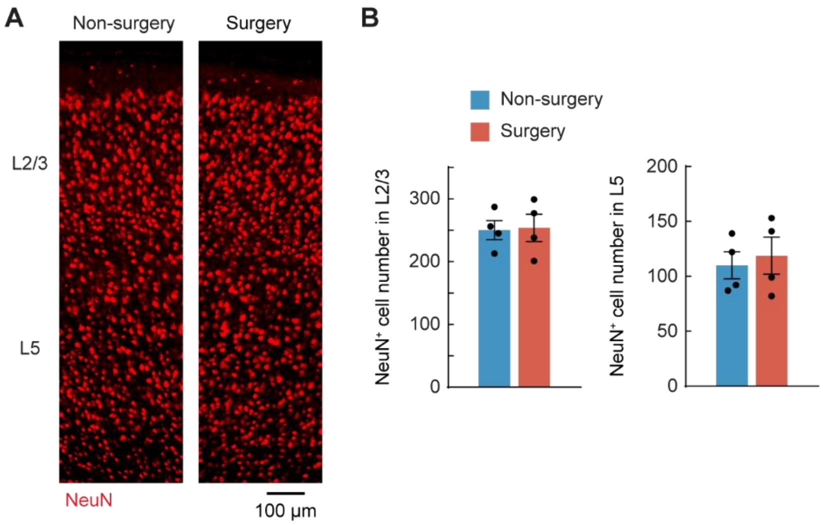

Figure S9. Surgery does not cause neuronal death in the mouse cortex at 24 h.
(**A**) Representative coronal sections of the mouse cortex stained with the neuronal marker NeuN (red). (**B**) Quantification of the data shown in **A** (*n* = 4 mice per group). Each dots represent data from a single mouse. Summary data are presented as mean ± s.e.m. Mann–Whitney test was used. See also **Table S1**.

Table S1. Details of statistical analysis.

**Figure 1**			
Fig. 1B	Group (# animals)		
2 d post-surgery	One-way ANOVA followed by Bonferroni's *post-hoc* test	F (2, 18) = 13.50	P = 0.0003
	Non-surgery (9) vs. anesthesia (4)	P > 0.9999	
	Non-surgery (9) vs. surgery (8)	P = 0.0005	
	Anesthesia (4) vs. surgery (8)	P = 0.0033	
7 d post-surgery	Mann–Whitney test (two-tailed)		
	Non-surgery (5) vs. surgery (4)	P = 0.0017	
Fig. 1E	One-way ANOVA followed by Bonferroni's *post-hoc* test	F (2, 15) = 34.38	P < 0.0001
2 d post-surgery	Non-surgery (7) vs. anesthesia (4)	P = 0.9471	
	Non-surgery (7) vs. surgery (7)	P < 0.0001	
	Anesthesia (4) vs. surgery (7)	P < 0.0001	
7 d post-surgery	Mann–Whitney test (two-tailed)		
	Non-surgery (5) vs. surgery (6)	P = 0.0325	
Fig. 1H	Mann–Whitney test (two-tailed)		
2 d post-surgery	Non-surgery (8) vs. surgery (8)	P = 0.003	
7 d post-surgery	Mann–Whitney test (two-tailed)		
	Non-surgery (8) vs. surgery (8)	P = 0.0207	
Fig. 1J	Mann–Whitney test (two-tailed)		
Rotarod	Non-surgery (7) vs. surgery (7)	P = 0.0175	
Treadmill	Mann–Whitney test (two-tailed)		
	Non-surgery (6) vs. surgery (6)	P = 0.0392	
T maze	Mann–Whitney test (two-tailed)		
	Non-surgery (7) vs. surgery (7)	P = 0.007	
**Figure 2**			
Fig. 2E	One-way ANOVA followed by Bonferroni's *post-hoc* test	F (2, 504) = 0.4132	P = 0.6618
Apical tuft	Non-surgery (184 dendrites from 6 mice) vs. anesthesia (140 dendrites from 4 mice)	P > 0.9999	
	Non-surgery vs. surgery (183 dendrites from 5 mice)	P > 0.9999	
	Anesthesia vs. surgery	P > 0.9999	
Trunk	One-way ANOVA followed by Bonferroni's *post-hoc* test	F (2, 626) = 2.883	P = 0.0567
	Non-surgery (232 trunks from 6 mice) vs. anesthesia (167 trunks from 4 mice)	P > 0.9999	
	Non-surgery vs. surgery (230 trunks from 5 mice)	P = 0.0608	
	Anesthesia vs. surgery	P = 0.6235	
L5 soma	One-way ANOVA followed by Bonferroni's *post-hoc* test	F (2, 515) = 0.5564	P = 0.5736
	Non-surgery (199 somas from 6 mice) vs. anesthesia (137 somas from 4 mice)	P > 0.9999	
	Non-surgery vs. surgery (182 somas from 5 mice)	P = 0.0008	

	Anesthesia vs. surgery	*P* = 0.0009	
Fig. 2F	One-way ANOVA followed by Bonferroni's *post-hoc* test	*F* (2, 504) = 20.22	*P* < 0.0001
Apical tuft	Non-surgery (184 dendrites from 5 mice) vs. anesthesia (140 dendrites from 4 mice)	*P* > 0.9999	
	Non-surgery vs. surgery (183 dendrites from 5 mice)	*P* < 0.0001	
	Anesthesia vs. surgery	*P* < 0.0001	
Trunk	One-way ANOVA followed by Bonferroni's *post-hoc* test	*F* (2, 622) = 54.13	*P* < 0.0001
	Non-surgery (228 trunks from 6 mice) vs. anesthesia (167 trunks from 4 mice)	*P* > 0.9999	
	Non-surgery vs. surgery (230 trunks from 5 mice)	*P* < 0.0001	
	Anesthesia vs. surgery	*P* < 0.0001	
L5 soma	One-way ANOVA followed by Bonferroni's *post-hoc* test	*F* (2, 479) = 52.02	*P* < 0.0001
	Non-surgery (199 soma from 6 mice) vs. anesthesia (137 soma from 4 mice)	*P* = 0.5155	
	Non-surgery vs. surgery (146 soma from 5 mice)	*P* < 0.0001	
	Anesthesia vs. surgery	*P* < 0.0001	
Fig. 2G	Mann–Whitney test (two-tailed)		
Apical tuft	Non-surgery (170 dendrites from 5 mice) vs. surgery (165 dendrites from 5 mice)	*P* < 0.0001	
Trunk	Non-surgery (174 trunks from 5 mice) vs. surgery (177 trunks from 5 mice)	*P* = 0.0234	
L5 soma	Non-surgery (167 soma from 5 mice) vs. surgery (99 soma from 3 mice)	*P* = 0.0008	
**Figure 3**			
Fig. 3C	Mann–Whitney test (two-tailed)		
Neutrophil	Non-surgery (6) vs. surgery (7)	*P* = 0.0693	
Ly6C^{hi} Mo	Non-surgery (6) vs. surgery (7)	*P* = 0.0373	
Ly6C^{low} Mo	Non-surgery (6) vs. surgery (7)	*P* = 0.0606	
F4/80^{hi} Mo	Non-surgery (6) vs. surgery (7)	*P* = 0.0397	
Fig. 3E	One-way ANOVA followed by Bonferroni's *post-hoc* test	*F* (2, 568) = 23.16	*P* < 0.0001
Treadmill ON/OFF	IgG2a + surgery (189 somas from 5 mice) vs. Anti-Ly6G + surgery (192 somas from 5 mice)	*P* = 0.9412	
	IgG2a + surgery vs. Anti-Gr1 + surgery (190 somas from 5 mice)	*P* < 0.0001	
	Anti-Ly6G + surgery vs. Anti-Gr1 + surgery	*P* < 0.0001	
Fig. 3F	One-way ANOVA followed by Bonferroni's *post-hoc* test	*F* (2, 10) = 6.982	*P* = 0.0127
Treadmill	IgG2a + surgery (4) vs. Anti-Ly6G + surgery (4)	*P* > 0.9999	
	IgG2a + surgery (4) vs. Anti-Gr + surgery (5)	*P* = 0.0309	
	Anti-Ly6G + surgery (4) vs. Anti-Gr1 + surgery (5)	*P* = 0.0285	
Fig. 3G	One-way ANOVA followed by Bonferroni's *post-hoc* test	*F* (2, 11) = 18.84	*P* =

			0.0003
Rotarod	IgG2a + surgery (4) vs. Anti-Ly6G + surgery (5)	$P > 0.9999$	
	IgG2a + surgery (4) vs. Anti-Gr1 + surgery (5)	$P = 0.0008$	
	Anti-Ly6G + surgery (5) vs. Anti-Gr1 + surgery (5)	$P = 0.0009$	
Fig. 3I	Mann–Whitney test (two-tailed)		
Treadmill ON/OFF	Vehicle + surgery (167 somas from 4 mice) vs. Clodronate + surgery (193 somas from 5 mice)	$P < 0.0001$	
Fig. 3J	Mann–Whitney test (two-tailed)		
Treadmill	Vehicle + surgery (6) vs. Clodronate + surgery (6)	$P = 0.0065$	
Fig. 3K	Mann–Whitney test (two-tailed)		
Rotarod	Vehicle (5) + surgery vs. Clodronate + surgery (6)	$P = 0.0043$	
Fig. 3N	One-way ANOVA followed by Bonferroni's *post-hoc* test	F (2, 16) = 7.272	P = 0.0057
T maze	Surgery (7) vs. surgery + Anti-Gr1 (6)	$P = 0.0182$	
	Surgery (7) vs. surgery + Clodronate (6)	$P = 0.0121$	
	Surgery + Anti-Gr1 (6) vs. surgery + Clodronate (6)	$P > 0.9999$	
**Figure 4**			
Fig. 4A	One-way ANOVA followed by Bonferroni's *post-hoc* test	F (4, 15) = 6.347	P = 0.0034
Plasma IL-1β	0 h (4) vs. 6 h (4)	$P = 0.0483$	
	0 h (4) vs. 24 h (4)	$P = 0.0061$	
	0 h (4) vs. 48 h (4)	$P = 0.1338$	
	0 h (4) vs. 168 h (4)	$P > 0.9999$	
Fig. 4B	One-way ANOVA followed by Bonferroni's *post-hoc* test	F (4, 15) = 7.267	P = 0.0018
Cortex	0 h (4) vs. 6 h (4)	$P = 0.0044$	
	0 h (4) vs. 24 h (4)	$P = 0.0258$	
	0 h (4) vs. 48 h (4)	$P = 0.6081$	
	0 h (4) vs. 168 h (4)	$P > 0.9999$	
Fig. 4C	Student's *t* test (two-tailed)		
Cortex	Non-surgery (3) vs. surgery (3)	$P = 0.0471$	
Fig. 4D	One-way ANOVA followed by Bonferroni's *post-hoc* test	F (2, 6) = 8.880	P = 0.0161
Cortex	IgG2a + surgery (3) vs. Anti-Ly6G + surgery (3)	$P > 0.9999$	
	IgG2a + surgery (3) vs. Anti-Gr1 + surgery (3)	$P = 0.0414$	
	Anti-Ly6G + surgery (3) vs. Anti-Gr1 + surgery (3)	$P = 0.026$	
Fig. 4E	Student's *t* test (two-tailed)		
Cortex	Vehicle + surgery (4) vs. clodronate + surgery (4)	$P = 0.0198$	
**Figure 5**			
Fig. 5C	Mann–Whitney test (two-tailed)		
Treadmill ON/OFF	Saline (216 somas from 5 mice) vs. IL-1β (224 somas from 5 mice)	$P < 0.0001$	
Fig. 5D	Mann–Whitney test (two-tailed)		
Treadmill	Saline (6) vs. IL-1β (4)	$P = 0.0095$	

Fig. 5E	Mann–Whitney test (two-tailed)		
Rotarod	Saline (6) vs. IL-1β (4)	P = 0.0381	
Fig. 5H	Mann–Whitney test (two-tailed)		
Treadmill ON/OFF	WT + surgery (171 somas from 5 mice) vs. $IL\text{-}1\beta^{-/-}$ + surgery (260 somas from 5 mice)	P < 0.0001	
Fig. 5I	Mann–Whitney test (two-tailed)		
Treadmill	WT + surgery (5) vs. $IL\text{-}1\beta^{-/-}$ + surgery (5)	P = 0.0159	
Fig. 5J	Mann–Whitney test (two-tailed)		
Rotarod	WT + surgery (5) vs. $IL\text{-}1\beta^{-/-}$ + surgery (5)	P = 0.0159	
Fig. 5M	One-way ANOVA followed by Bonferroni's *post-hoc* test	F (2, 532) = 11.81	P < 0.0001
Treadmill ON/OFF	$CD11b^{-}$ (WT) (175 somas from 4 mice) vs. $CD11b^{+}$ (WT) (175 somas from 5 mice)	P = 0.0381	
	$CD11b^{-}$ (WT) vs. $CD11b^{+}$ ($IL\text{-}1\beta^{-/-}$) (185 somas from 5 mice)	P = 0.6934	
	$CD11b^{+}$ (WT) vs. $CD11b^{+}$ ($IL\text{-}1\beta^{-/-}$)	P = 0.0013	
Fig. 5N	One-way ANOVA followed by Bonferroni's *post-hoc* test	F (2, 17) = 5.286	P = 0.0164
Treadmill	$CD11b^{-}$ (WT) (6) vs. $CD11b^{+}$ (WT) (8)	P = 0.0365	
	$CD11b^{-}$ (WT) (6) vs. $CD11b^{+}$ ($IL\text{-}1\beta^{-/-}$) (6)	P > 0.9999	
	$CD11b^{+}$ (WT) (8) vs. $CD11b^{+}$ ($IL\text{-}1\beta^{-/-}$) (6)	P = 0.0468	
Fig. 5O	One-way ANOVA followed by Bonferroni's *post-hoc* test	F (2, 15) = 6.107	P = 0.0115
Rotarod	$CD11b^{-}$ (WT) (5) vs. $CD11b^{+}$ (WT) (7)	P = 0.0228	
	$CD11b^{-}$ (WT) (5) vs. $CD11b^{+}$ ($IL\text{-}1\beta^{-/-}$) (6)	P > 0.9999	
	$CD11b^{+}$ (WT) (7) vs. $CD11b^{+}$ ($IL\text{-}1\beta^{-/-}$) (6)	P = 0.0377	
**Figure 6**			
Fig. 6A	Mann–Whitney test (two-tailed)		
NLRP3	Non-surgery (4) vs. surgery (6)	P = 0.019	
Cleaved casp-1	Non-surgery (4) vs. surgery (6)	P = 0.0095	
ASC	Non-surgery (4) vs. surgery (6)	P = 0.0301	
Fig. 6B	Mann–Whitney test (two-tailed)		
ASC&$Cx3Cr1^{GFP}$	Non-surgery (4) vs. surgery (4)	P = 0.8286	
Fig. 6D	Mann–Whitney test (two-tailed)		
$CD11b^{+}$ IL-1β	Saline + surgery (4) vs. MCC950 + surgery (4)	P = 0.0286	
Fig. 6E	Mann–Whitney test (two-tailed)		
Cortex IL-1β	Saline + surgery (4) vs. MCC950 + surgery (4)	P = 0.0285	
Fig. 6F	Mann–Whitney test (two-tailed)		
Treadmill OFF	Saline + surgery (185 somas from 4 mice) vs. MCC950 + surgery (230 somas from 6 mice)	P = 0.8231	
Treadmill ON/OFF	Saline + surgery (161 somas from 4 mice) vs. MCC950 + surgery (192 somas from 6 mice)	P < 0.0001	
Fig. 6G	Mann–Whitney test (two-tailed)		
Treadmill	Saline + surgery (4) vs. MCC950 + surgery (6)	P = 0.0317	
Fig. 6H	Mann–Whitney test (two-tailed)		

Rotarod	Saline + surgery (4) vs. MCC950 + surgery (6)	P = 0.019	
**Figure 7**			
Fig. 7B	Mann–Whitney test (two-tailed)		
Formation	Non-surgery (4) vs. surgery (4)	P = 0.6571	
Elimination	Non-surgery (4) vs. surgery (4)	P = 0.0571	
Fig. 7C	Mann–Whitney test (two-tailed)		
Formation	Non-surgery (4) vs. surgery (4)	P = 0.6571	
Elimination	Non-surgery (4) vs. surgery (4)	P = 0.3429	
Fig. 7E Left	One-way ANOVA followed by Bonferroni's *post-hoc* test	F (5, 18) = 14.39	P < 0.0001
Formation	Non-surgery (4) vs. surgery (4)	P = 0.0009	
	Non-surgery (4) vs. IL-1β (4)	P = 0.0018	
	Non-surgery (4) vs. surgery + vehicle liposomes (4)	P = 0.0012	
	Non-surgery (4) vs. surgery + clodronate liposomes (4)	P = 0.9999	
	Surgery (4) vs. IL-1β (4)	P = 0.9999	
	Surgery (4) vs. $IL\text{-}1\beta^{-/-}$ + surgery (4)	P = 0.0003	
	Surgery vs. surgery + clodronate liposomes (4)	P = 0.013	
	IL-1β vs. surgery + vehicle liposomes (4)	P = 0.9999	
	IL-1β vs. surgery + clodronate liposomes (4)	P = 0.0287	
	Surgery + vehicle liposomes (4) vs. surgery + clodronate liposomes (4)	P = 0.019	
Fig. 7E Right	One-way ANOVA followed by Bonferroni's *post-hoc* test	F (5, 18) = 2.965	P = 0.0400
Elimination	Non-surgery (4) vs. surgery (4)	P = 0.9999	
	Non-surgery (4) vs. IL-1β (4)	P = 0.4496	
	Non-surgery (4) vs. surgery + vehicle liposomes (4)	P = 0.1862	
	Non-surgery (4) vs. surgery + clodronate liposomes (4)	P = 0.9999	
	Surgery (4) vs. IL-1β (4)	P = 0.9999	
	Surgery (4) vs. $IL\text{-}1\beta^{-/-}$ + surgery (4)	P = 0.9999	
	Surgery vs. surgery + clodronate liposomes (4)	P = 0.9999	
	IL-1β vs. surgery + vehicle liposomes (4)	P = 0.9999	
	IL-1β vs. surgery + clodronate liposome (4)	P = 0.3671	
	Surgery + vehicle liposomes (4) vs. surgery + clodronate liposomes (4)	P = 0.1507	
Fig. 7F	One-way ANOVA followed by Bonferroni's *post-hoc* test	F (5, 18) = 15.63	P < 0.0001
Net change	Non-surgery (4) vs. surgery (4)	P = 0.0006	
	Non-surgery (4) vs. IL-1β (4)	P = 0.0005	
	Non-surgery (4) vs. surgery + vehicle liposomes (4)	P = 0.0002	
	Non-surgery (4) vs. surgery + clodronate liposomes (4)	P = 0.9999	
	Surgery (4) vs. IL-1β (4)	P = 0.9999	
	Surgery (4) vs. $IL\text{-}1\beta^{-/-}$ + surgery (4)	P = 0.0017	
	Surgery vs. surgery + clodronate liposomes (4)	P = 0.0069	
	IL-1β vs. surgery + vehicle liposomes (4)	P = 0.9999	
	IL-1β vs. surgery + clodronate liposomes (4)	P = 0.0052	

	Surgery + vehicle liposomes (4) vs. surgery + clodronate liposomes (4)	*P* = 0.0024	
**Figure S1**			
Fig. S1B	One-way ANOVA followed by Bonferroni's *post-hoc* test	*F* (2, 12) = 0.06373	*P* = 0.9388
Grooming	Non-surgery (5) vs. anesthesia (5)	*P* > 0.9999	
	Non-surgery (5) vs. surgery (5)	*P* > 0.9999	
	Anesthesia (5) vs. surgery (5)	*P* > 0.9999	
Fig. S1C	One-way ANOVA followed by Bonferroni's *post-hoc* test	*F* (2, 17) = 0.7831	*P* = 0.4728
Total distance	Non-surgery (7) vs. anesthesia (5)	*P* > 0.9999	
	Non-surgery (7) vs. surgery (8)	*P* > 0.9999	
	Anesthesia (5) vs. surgery (8)	*P* = 0.6933	
Time in the center	One-way ANOVA followed by Bonferroni's *post-hoc* test	*F* (2, 17) = 0.6081	*P* = 0.5558
	Non-surgery (7) vs. anesthesia (5)	*P* = 0.9298	
	Non-surgery (7) vs. surgery (8)	*P* > 0.9999	
	Anesthesia (5) vs. surgery (8)	*P* > 0.9999	
Average speed	One-way ANOVA followed by Bonferroni's *post-hoc* test	*F* (2, 17) = 0.7831	*P* = 0.4728
	Non-surgery (7) vs. anesthesia (5)	*P* > 0.9999	
	Non-surgery (7) vs. surgery (8)	*P* > 0.9999	
	Anesthesia (5) vs. surgery (8)	*P* = 0.6933	
**Figure S2**			
Fig. S2A	Mann–Whitney test (two-tailed)		
Young 2 d	Non-surgery (8) vs. surgery (8)	*P* = 0.1069	
Young 7 d	Non-surgery (8) vs. surgery (8)	*P* = 0.0963	
Aged 2 d	Non-surgery (7) vs. surgery (7)	*P* = 0.0533	
Fig. S2B	One-way ANOVA followed by Bonferroni's *post-hoc* test	*F* (2, 18) = 1.094	*P* = 0.3560
Aged	Surgery (7) vs. Anti-Gr1 (7)	*P* = 0.4985	
	Surgery (7) vs. Clodronate (7)	*P* = 0.9862	
	Anti-Gr1 (7) vs. Clodronate (7)	*P* > 0.9999	
**Figure S4**			
Fig. S4A	Mann–Whitney test (two-tailed)		
Frequency	Non-surgery (5) vs. surgery (5)	*P* < 0.0001	
Fig. S4B	Mann–Whitney test (two-tailed)		
Amplitude	Non-surgery (5) vs. surgery (5)	*P* = 0.7342	
Fig. S4D	Mann–Whitney test (two-tailed)		
Peak time	Non-surgery (5) vs. surgery (5)	*P* < 0.0001	
**Figure S5**			
Fig. S5C	Mann–Whitney test (two-tailed)		
Iba1 density	Non-surgery (6) vs. surgery (8)	*P* = 0.277	
Iba1 soma size	Non-surgery (4) vs. surgery (4)	*P* = 0.681	
Iba1 soma MFI	Non-surgery (4) vs. surgery (4)	*P* = 0.1499	

Iba1 branch	Non-surgery (4) vs. surgery (4)	*P* = 0.5946	
Fig. S5E	Mann–Whitney test (two-tailed)		
Border	Non-surgery (8) vs. surgery (8)	*P* = 0.817	
Parenchyma	Non-surgery (8) vs. surgery (8)	*P* > 0.9999	
Fig. S5G	Mann–Whitney test (two-tailed)		
B cells	Non-surgery (6) vs. surgery (6)	*P* = 0.5714	
T cells	Non-surgery (6) vs. surgery (6)	*P* = 0.8983	
**Figure S6**			
Fig. S6B	Student's *t* test (two-tailed)		
$Ly6C^{low}$	Vehicle (3) vs. clodronate (3)	*P* = 0.0130	
$Ly6C^{hi}$	Vehicle (3) vs. clodronate (3)	*P* = 0.0047	
Fig. S6D	Mann–Whitney test (two-tailed)		
Cortex	Vehicle (4) vs. clodronate (4)	*P* = 0.9714	
Spleen	Vehicle (4) vs. clodronate (4)	*P* = 0.0286	
Liver	Vehicle (4) vs. clodronate (4)	*P* = 0.0286	
**Figure S7**			
Fig. S7A	One-way ANOVA followed by Bonferroni's *post-hoc* test	*F* (4, 15) = 7.480	*P* = 0.0016
Plasma TNF-α	0 h (4) vs. 6 h (4)	*P* = 0.0022	
	0 h (4) vs. 24 h (4)	*P* > 0.9999	
	0 h (4) vs. 48 h (4)	*P* > 0.9999	
	0 h (4) vs. 168 h (4)	*P* > 0.9999	
Fig. S7B	One-way ANOVA followed by Bonferroni's *post-hoc* test	*F* (4, 15) = 0.03234	*P* = 0.9978
Cortex TNF-α	0 h (4) vs. 6 h (4)	*P* > 0.9999	
	0 h (4) vs. 24 h (4)	*P* > 0.9999	
	0 h (4) vs. 48 h (4)	*P* > 0.9999	
	0 h (4) vs. 168 h (4)	*P* > 0.9999	
Fig. S7C	One-way ANOVA followed by Bonferroni's *post-hoc* test	*F* (4, 15) = 3.329	*P* = 0.0386
Plasma IL-6	0 h (4) vs. 6 h (4)	*P* = 0.0306	
	0 h (4) vs. 24 h (4)	*P* > 0.9999	
	0 h (4) vs. 48 h (4)	*P* > 0.9999	
	0 h (4) vs. 168 h (4)	*P* > 0.9999	
Fig. S7D	One-way ANOVA followed by Bonferroni's *post-hoc* test	*F* (4, 15) = 0.01971	*P* = 0.9991
Cortex IL-6	0 h (4) vs. 6 h (4)	*P* > 0.9999	
	0 h (4) vs. 24 h (4)	*P* > 0.9999	
	0 h (4) vs. 48 h (4)	*P* > 0.9999	
	0 h (4) vs. 168 h (4)	*P* > 0.9999	
**Figure S9**			
Fig. S9C	Mann–Whitney test (two-tailed)		
L2/3	Non-surgery (4) vs. surgery (4)	*P* > 0.9999	
L5	Non-surgery (4) vs. surgery (4)	*P* = 0.6571	
